# Detection of HIV-1 and Human Proteins in Urinary Extracellular Vesicles from HIV+ Patients

**DOI:** 10.1155/2018/7863412

**Published:** 2018-03-12

**Authors:** Samuel I. Anyanwu, Akins Doherty, Michael D. Powell, Chamberlain Obialo, Ming B. Huang, Alexander Quarshie, Claudette Mitchell, Khalid Bashir, Gale W. Newman

**Affiliations:** ^1^Department of Microbiology, Biochemistry and Immunology, Morehouse School of Medicine, Atlanta, GA, USA; ^2^Department of Medicine, Morehouse School of Medicine, Atlanta, GA, USA; ^3^Clinical Research Center, Morehouse School of Medicine, Atlanta, GA, USA

## Abstract

**Background:**

Extracellular vesicles (EVs) are membrane bound, secreted by cells, and detected in bodily fluids, including urine, and contain proteins, RNA, and DNA. Our goal was to identify HIV and human proteins (HPs) in urinary EVs from HIV+ patients and compare them to HIV− samples.

**Methods:**

Urine samples were collected from HIV+ (*n* = 35) and HIV− (*n* = 12) individuals. EVs were isolated by ultrafiltration and characterized using transmission electron microscopy, tandem mass spectrometry (LC/MS/MS), and nanoparticle tracking analysis (NTA). Western blots confirmed the presence of HIV proteins. Gene ontology (GO) analysis was performed using FunRich and HIV Human Interaction database (HHID).

**Results:**

EVs from urine were 30–400 nm in size. More EVs were in HIV+ patients, *P* < 0.05, by NTA. HIV+ samples had 14,475 HPs using LC/MS/MS, while only 111 were in HIV−. HPs in the EVs were of exosomal origin. LC/MS/MS showed all HIV+ samples contained at least one HIV protein. GO analysis showed differences in proteins between HIV+ and HIV− samples and more than 50% of the published HPs in the HHID interacted with EV HIV proteins.

**Conclusion:**

Differences in the proteomic profile of EVs from HIV+ versus HIV− samples were found. HIV and HPs in EVs could be used to detect infection and/or diagnose HIV disease syndromes.

## 1. Introduction

Extracellular vesicles (EVs) are membrane bound vesicles, between 30 nm and 1 *μ*m in size, are secreted into blood, urine, saliva, semen, and other bodily fluids, and have been suggested as a potential source of biomarkers for disease progression [1, 2]. These EVs, microparticles and/or exosomes, are secreted by cells normally or while they are undergoing stress or apoptosis [3] and contain proteins, mRNA, and miRNA [4] that are involved in cell to cell communication, transfer of antigens to cells, and intracellular communication. EVs are described in cancer disease pathogenesis [5] in HIV infection [6], other viral infections [7], and other disease states such as cardiovascular, renal, liver, and metabolic disease [8–11].

EVs from urine are an attractive noninvasive source for biomarkers of diseases [12, 13]. In healthy individuals, protein only accounts for 0.01% of urine components; however, in certain disease states, the protein content and EV numbers can increase in urine [12–16]. The glomerular capsule filters blood that is passed into the renal tubule and accounts for thirty percent of the urinary protein content [14–16]. The remaining seventy percent of proteins in urine is derived from the kidney [17], and thus, urinary EVs are comprised of both renal and efferent components.

HIV proteins are detected in EVs of HIV+ patients and HIV Nef is the most prevalent protein found [18–21]. Other reports of HIV proteins in EVs are from* in vitro* transfected or HIV infected cultured cells and are not from HIV+ patient samples [6, 18, 19, 22, 23].

Biomarkers in urinary EVs are suggested for use in the diagnosis of many disease states [12, 13, 24–30]. The objectives of this study were to determine the differences in proteins from urinary EVs from HIV+ patients and HIV− individuals using proteomics and mass spectrometry. The analysis of more patient samples could identify specific EV urinary proteins as biomarkers of HIV infection, treatment efficacy, and/or disease progression.

## 2. Methods

### 2.1. Sample Collection

Urine was collected from thirty-five (35) HIV+ patients and twelve (12) HIV− individuals in sterile collection cups. The subjects were recruited from clinics in the metropolitan Atlanta area, GA. Patient demographics are described in Table 1. The study was approved by the Institutional Review Board of Morehouse School of Medicine and written informed consent was obtained from all participants.

### 2.2. EV Isolation

Urine samples were centrifuged at 1000 ×g to remove cells and sediment then frozen at −80°C. Samples, 4 ml, were thawed and the EVs isolated followed by centrifugal filtration using Amicon Ultra-4 100 kDa centrifugal filter unit (Millipore, Billerica, MA), at 3000 ×g for 15 minutes at 4°C. The retentate, containing EVs, was collected from the top of the filter and resuspended in 200 *μ*l phosphate buffered saline (PBS) for use in the transmission electron microscopy and tandem mass spectrometry (LC/MS/MS) analysis.

### 2.3. Transmission Electron Microscopy Analysis

Transmission electron microscopy (TEM) was used to identify EVs in two HIV-1 positive and two HIV-1 negative samples. Urinary EVs were fixed in 2.5% glutaraldehyde in 0.1 M cacodylate buffer for 2 hours at 4°C followed by 2 washes with 0.1 M cacodylate buffer, 5 minutes each. Samples were stained with 1% osmium tetroxide in 0.1 M cacodylate buffer for 1 hour at 4°C followed by 2 washes with the cacodylate buffer and 3 washes with deionized water, 5 minutes each. Samples were subsequently stained with 0.5% aqueous uranyl acetate for 2 hours at room temperature and subsequently viewed with a JEOL 1200EX transmission electron microscope (JEOL, Peabody, MA).

### 2.4. Nanoparticle Tracking Analysis (NTA)

Urine samples from HIV-negative (*n* = 8) and positive individuals (*n* = 11), 15 ml, were centrifuged at 300 ×g for 10 min at 4°C to remove cell debris. The supernatant was collected and centrifuged at 16,500 ×g for 20 min at 4°C and the supernatant collected and ultracentrifuged at 120,000 ×g at 4°C for 1.5 hr. The pellet was resuspended in 500 *μ*l of PBS. The size and quantification of the EVs were analyzed using the NanoSight NS500 (NanoSight NTA 2.3 Nanoparticle Tracking and Analysis Release version build 0025). Particles were automatically tracked and sized based on Brownian motion and the diffusion coefficient. The NTA measurement conditions were temperature 21.0 +/− 0.5°C, viscosity 0.99 +/− 0.01 cP, frames per second 24.99–25, and measurement time 30 s. The detection threshold was similar in all samples. Two recordings were performed for each sample.

### 2.5. Mass Spectrometry Analysis

Thirty-five (35) HIV+ and twelve (12) HIV− EV samples were lysed and trypsinized and the sequence of peptides was determined by tandem mass spectrometry (LC/MS/MS), using an LTQ Ion Trap Mass Spectrometer (Thermo Fischer Scientific, Waltham, MA). Peptides were first reduced in DTT 10 mM at 56°C for at least 30 min and alkylated with 15 mM iodoacetic acid for 30 min at room temperature in the dark. Samples were then digested with mass spectrometry grade trypsin 20 ng/*μ*l for 4 hours at 37°C. Just before analysis, the sample was acidified by the addition of formic acid to 0.1%. Peptides were separated by reverse phase HPLC (Agilent) on a 0.5 × 75 mm C-18 column (Michrom) at a flow rate of 500 nl/min using a linear gradient of acetonitrile (5–35%) over 100 min. Ions were directly introduced by nanospray and spectra were collected using Xcalibur 2.0 software using an intensity threshold of 200 counts. The resulting spectra were analyzed using Bioworks 1.1 software to search a hybrid Human-HIV database created from the complete nonredundant peptide database from NCBI. The threshold for inclusion in the search is a minimal S/N ratio of 3. False discovery rates were determined and set based on the control HIV− samples. An initial protein identification list was generated from matches with an Xcorr score versus charge state of 1.0 (+1) 1.5 (+2) and 1.7 (+3) and consensus scores greater than 10.0.

Bioinformatics techniques for analysis of HIV EV proteins were used on the LC/MS/MS detected proteins [31]. Functional enrichment analysis was performed using FunRich (Functional Enrichment analysis tool, http://funrich.org/index.html) [32] against a human database to detect proteins involved in biological processes, cellular components, sites of expression, and biological pathways. Only processes with a *P* value < 0.05, using the Benjamini-Hochberg False Discovery rate, were reported. The human proteins detected were compared to the top 100 EV proteins in ExoCarta (http://exocarta.org/exosome_markers_new) [33, 34], sixty EV proteins in the EV array [35], and proteins identified in EVs from HIV infected lymphocytic cells [36].

Pathway analysis comparing HIV+ samples with CD4+ T cells greater than 300 (*n* = 15) to those with less than 500 (*n* = 15) and HIV high VL, greater than 200 copies (*n* = 10), compared to HIV low viral loads, less than 200 copies (*n* = 10), was done using Pathway Studio version 11.4 Mammal Plus (Elsevier, Inc., Atlanta, GA). Gene Set Enrichment Analysis (GSEA) was used to identify the top 10 curated pathways for the proteins in the each of the patient groups. No comparisons were done between patients not on ART or undergoing ART because there was only one patient not on ART.

The HIV proteins, Nef, Vpr, Vpu, and Vif, were searched using the HIV-1 Human Interaction database (https://www.ncbi.nlm.nih.gov/genome/viruses/retroviruses/hiv-1/interactions/). This database contains all the known, published interactions of HIV-1 gene products with human proteins [37]. Proteins from the search were compared to the human proteins detected in the HIV EVs.

### 2.6. Western Blot Analysis

To validate the presence of HIV proteins in urinary EVs, western blot analysis (WB) was performed on twenty (20) randomly selected HIV+ and three (3) HIV− control urine samples. Recombinant HIV-1 Nef and HIV-1 p24 were used as positive controls, while HIV-negative urine and HIV-positive filtrate were used as negative controls. Samples were heated at 85°C for two minutes in a tris-glycine SDS sample buffer, were loaded into a 4–20% TGX gradient gel (Bio-Rad, Hercules, CA), and run for 50 mins at 200 V. A semidry transfer unit (Hoefer Scientific, Holliston, MA) was used to transfer the separated proteins onto a PVDF membrane (Bio-Rad) at 15 V for 50 mins. The filter was blocked for nonspecific binding using 5% nonfat dry milk in 1x tris buffered saline (TBS) with Tween 20. The membrane was incubated overnight in pooled plasma from twenty HIV+ patients as the primary antibody at a 1 : 500 dilution and rabbit anti-human IgG conjugated HRP antibody (1 : 1000, Bio-Rad, Hercules, CA) was used as secondary antibody. Super Signal West Femto (Thermo Fischer Scientific, Waltham, MA) was used as a chemiluminescent substrate for detection. The membrane was developed and imaged using the LAS 4000 biomolecular imager (GE Healthcare Life Sciences, Pittsburgh, PA). Recombinant HIV-1 Nef and p24 WB analyses were detected using anti-Nef and p24 monoclonal antibodies (1 : 500, EMD Millipore, Billerica, MA) and anti-mouse IgG conjugated HRP antibodies (1 : 1000, Bio-Rad, Hercules, CA) were used.

### 2.7. HIV p24 ELISA

Twenty-six (26) HIV+ and eleven (11) HIV− urine samples were tested for the presence of HIV p24 by ELISA (ImmunoDX, Woburn, MA).

## 3. Results

### 3.1. HIV Proteins Are Present in Urinary EVs of HIV-Positive Patients

LC/MS/MS mass spectrometry HIV EV protein results are presented in Table 2. Urinary EV proteins meeting the false discovery rate and Xcorr score criteria as HIV-1 proteins included Nef, Gag, Pol, Protease, gp120, gp160, gp41, Rev, reverse transcriptase, Tat, Vif, Vpr, and Vpu. All HIV+ urine samples (*n* = 35) contained at least one HIV-1 protein in EVs, while no HIV proteins were found in the HIV− samples (*n* = 12) (Table 3). HIV-1 Nef was detected in twenty-six of thirty-five (26 of 35) (74.3%) HIV+ urine samples. Three (3) patients' urine samples, #173, #174, and #196, were tested 203, 311, and 35 days, respectively, after their first EV sample was analyzed. No difference in the HIV proteins detected in sample #196, 35 days after his previous sample, was found. #173's sample, tested 203 days after the first analysis, had a similar profile, except that Rev and Tat were not detected. In addition, #174's EVs examined 311 days after the first sampling found Rev and RT missing from the profile.

HIV p24 antigen was only detected in five of thirty-five (5 of the 35 patient) (14%) samples by LC/MS/MS, but of the twenty-six (26) HIV+ and eleven (11) HIV-negative samples tested by ELISA, no p24 was detected. There was no statistical correlation of the number of HIV proteins detected with CD4+ T cell counts, viral loads, or ART therapy.

**Figure 1:**
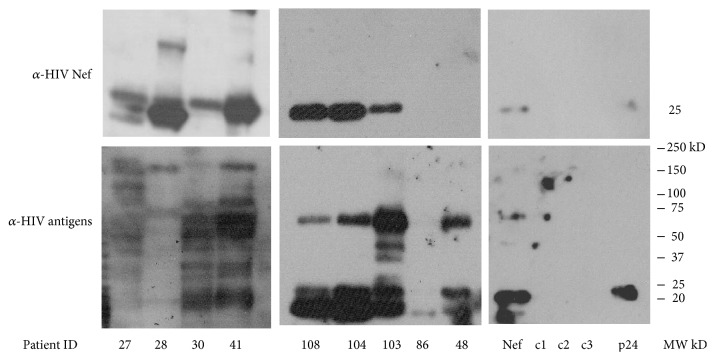
*Detection of HIV-1 proteins by western blot*. Extracellular vesicles were isolated from four ml of urine from HIV-1+ patients and HIV-1 negative individuals by Amicon ultrafiltration (MW cutoff = 100,000 kD). The western blot is representative of 9 HIV+ and 3 HIV-negative samples (c1, c2, and c3). Recombinant HIV Nef and p24 were added as positive controls (last panels on the right). Samples were isolated in a 4–20% gradient SDS gel and transferred to a PVDF membrane. The filter was incubated with the primary antibody, pooled HIV-1 positive plasma (bottom panels), or a monoclonal anti-HIV Nef (top panels). The secondary antibody, goat anti-mouse IgG for the anti-Nef blots or rabbit anti-human IgG for the anti-HIV antibodies, conjugated to horseradish peroxidase. Super Signal West Femto was used as chemiluminescent substrate for detection.

Validation by WB analysis using polyclonal pooled patient serum and monoclonal antibodies against HIV Nef and HIV p24 indicated the presence of HIV proteins.  Figure 1 is a WB using polyclonal pooled HIV+ serum used as the detection antibody. All the HIV+ patient samples contained HIV-1 proteins and the top panel shows patient samples reacting to anti-HIV Nef. HIV+ urine samples, 7 of 9 (77.7%), showed HIV-1 Nef bands at 27 kD.

### 3.2. TEM and NTA Analysis of EVs

**Figure 2:**
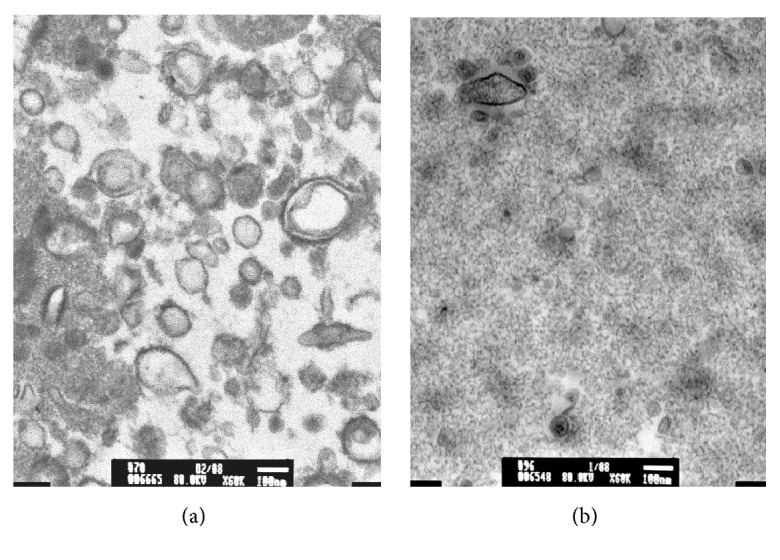
*Transmission electron microscopy of urinary extracellular vesicles*. Four mls of urine was used to isolate EVs by Amicon ultrafiltration (MW cutoff = 100,000 kD). EVs were fixed in 2.5% glutaraldehyde in 0.1 M cacodylate buffer. Samples were stained with 1% osmium tetroxide in 0.1 M cacodylate buffer and subsequently stained with 0.5% aqueous uranyl acetate. A JEOL 1200EX transmission electron microscope (JEOL, Peabody, MA) was used for observation and photography.** 1A**. EVs from HIV-1 posi.

**Figure 3:**
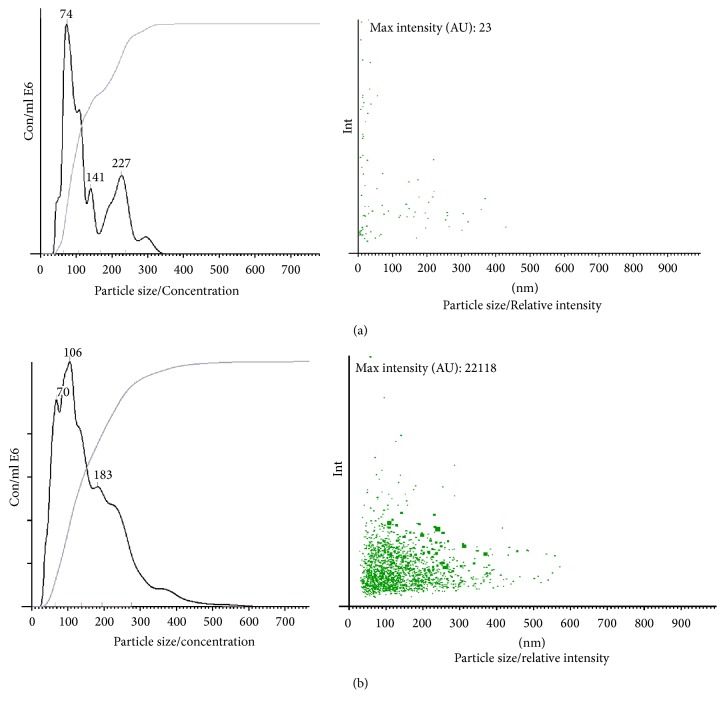
*Nanosight analysis* (representative analysis). (a) NTA analysis of an HIV-negative urine sample had 0.4 × 10^8^ particles per ml (left panel) while (b) depicts an urine sample from a HIV+ patient that had 8.7 × 10^8^ particles per ml and has a greater relative intensity profile (right panel (a) and (b)) when compared to the HIV-negative sample. The Rank Sum *T* test showed that HIV+ patient urine samples had more particles per ml than the negative control urine (*P* < 0.05).

TEM analysis of urine from HIV+ patients showed multiple EVs, ranging in size from 50 nm to 300 nm (Figure 2(a)), while two HIV-negative controls had fewer EVs present (Figure 2(b)). NTA analysis showed that there were significantly more EVs from HIV+ patients than healthy controls, 4.96 ± 0.0733 and 3.69 ± 0.075, respectively (*P* < 0.05). No significant differences were found in the size of the EVs, 110–227 nm for HIV-negative donors and 54–448 nm HIV+ samples. Representative Nanosight analyses for HIV-negative and HIV+ urine samples are shown in Figure 3.

### 3.3. Human Proteins in HIV+ and Negative EV Urine Samples

EV proteins from the HIV+ patients, 14,475, which entered into FunRich, functional enrichment analysis software, showed 29.44% or 1,932 proteins were associated with exosomes (Table 4). These EV identified proteins were compared to top 100 EV proteins in the ExoCarta database with 83% matching (http://exocarta.org/exosome_markers_new) [33], 22 EV proteins in the EV array [35] were similar, and 7 of 14 EV proteins identified in exosomes from HIV infected lymphocytes [36] were found and are highlighted in Table 4. Exosomal proteins found in the control samples are listed in Table 5.

**Figure 4:**
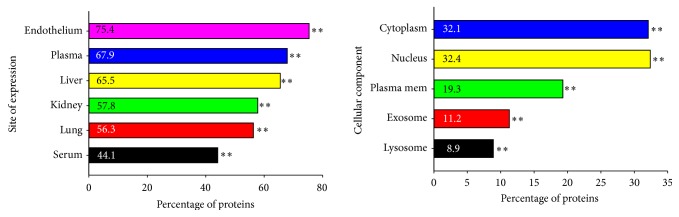
*Percentage of proteins found in HIV+ urinary EVs*. FunRich analysis of the LC/MS/MS proteins from HIV+ EVs determined the most likely tissue expressing the proteins, site of expression, and the cellular component from which the protein is derived. Data is graphed as the percentage of proteins found. *∗∗* denotes significance, *P* < 0.01.

The GO results of the FunRich analysis of the EVs from the HIV+ samples are summarized in Table 6 and Figure 4. The top five (*P* < 0.01) EV sites of expression were endothelial cells, plasma, liver, serum, and kidney and the most significant cellular components were lysosomes, exosomes, membranes, plasma membranes, the nucleus, and the cytoplasm (*P* < 0.01) (Figure 4). The top five ontologies (Table 6) were protein serine/threonine kinase activity, catalytic activity, GTPase activator activity, guanyl-nucleoside exchange factor activity, and cell adhesion molecule activity (*P* < 0.0001), the top biological process was regulation of nucleobase, nucleoside, and nucleic acid (*P* < 0,0001), and the most prominent biological pathway was integrin cell surface interactions (*P* < 0.03).

LC/MS/MS identified 15,571 proteins in EVs from HIV+ patients with CD4+ T cells greater than 300, 2,115 from CD4+ T cells less than 300, 15,028 proteins from patients with low VL, and 2486 from patients with high VLs. Pathway analysis was similar between EV proteins from patients with greater than 300 CD4+ T cells and low VLs and different between the low CD4+ T cells and high VLs (summarized in Table 7). The pathways found are detailed in Supplementary Material 1. Interleukin proteins detected were IL10, IL10RA, IL16, IL17RC, IL18, IL18BP, IL1RAP, IL1RL2, IL1RN, IL33, IL4I1, IL6, and IL6ST. Immunomodulatory molecules, HOXB4, CD81, CD9, TGF-*β*1, IDO, Notch1, ADAM17, Rab4, and HGF, were also found by LC/MS/MS in addition to MHC Class I and II antigens.

The HIV-1 Human Interaction database search found that HIV Nef interacted with 559 EV proteins of 770 total human proteins (72.6%); HIV Vpr interacted with 437 EV of 598 human (73.1%); HIV Vif interacted with 162 EV of 310 human (52.2%); and HIV Vpu interacted with 165 EV of 244 human proteins which were found in the HIV+ EVs (67.6%) (see Supplementary Material 2, including PMIDs for references).

Functional analysis of the control EVs are listed in Table 8. The major sites of expression were cervicovaginal fluid, neutrophils, and gastric juice (*P* < 0.0001). The most significant ontologies were molecular function of the proteins and defense/immunity protein activity and principal biological processes were immune response, signal transduction, cell communication, and antigen presentation (*P* < 0.0073).

Only sixty-four (64) proteins overlapped between the HIV+ and control EV samples and are listed in Table 8. The top fourteen (14) GO ontologies for cellular components include extracellular exosome, extracellular region, extracellular space, hemoglobin complex, and blood microparticle (*P* < 0.001, Table 9), GO ontologies for molecular function were heparin binding, ion gated activity, and oxygen transporter activity, and the most significant biological processes found were response to yeast, defense response to fungus, macrophage chemotaxis, negative regulation of growth of symbiont in host, oxygen transport, and hydrogen peroxide catabolic process.

## 4. Discussion

This is the first report of the detection of urinary EVs containing HIV and human proteins from HIV+ patients by mass spectrometry and western blot. EVs provide intercellular communication to cells through the delivery of their cargo, nucleic acids, miRNAs, and proteins, to recipient cells reviewed in [3]. Previous studies have found EVs in plasma of HIV+ patients but did not describe HIV or human proteins within them. Others have described EVs containing HIV proteins but these results were from* in vitro* HIV infected cell cultures and not from HIV+ patients [18, 20, 22, 23, 36, 38–47]. This study details both the HIV and human proteins found in urinary EVs from HIV+ patients.

According to the International Society for Extracellular Vesicles (ISEV), the minimal requirements for EVs or their presence in samples includes the simultaneous detection of transmembrane proteins and cytosolic proteins with membrane/receptor binding abilities, while major cell organelles are absent [48]. LC/MS/MS analysis identified these proteins and functional enrichment analysis determined a significant number which were of exosomal origin in both the EVs in HIV+, 1,932, and HIV−, only 37. TEM analysis of HIV+ and HIV− urine showed pleiotropic membrane bound vesicles in both groups' urine samples and NTA analysis showed particles ranging in size from 50 nm to 300 nm in both groups, although the HIV+ samples had significantly more particles than uninfected samples. Other studies have found increased numbers of EVs in the plasma of HIV+ patients [43, 49]. Proteins from both the HIV+ and HIV− individuals were significantly associated with exosomal proteins, further substantiating our hypothesis that urine from HIV+ patients contains EVs (Table 10). The FunRich analysis of the sites of expression showed that a significant number of proteins were associated with the endothelium, plasma, serum, kidney, liver, and lung. These findings suggest that EVs from HIV+ patients may be filtered from these sites and concentrated in urine.

HIV has previously been detected in the urine of HIV+ patients; however, it was shown that HIV virions are associated with cell pellets and not in centrifuged urine [50, 51]. p24 is found in replicative HIV infectious virions but was not found in twenty-six of our HIV+ samples by ELISA and only five of thirty-five HIV+ EV urine samples had detectable p24 by LC/MS/MS analysis. p24 in urine pellets is derived from mononuclear cells but was found in only 3 of 80 analyzed samples [51]. This represents a low sensitivity, primarily because the HIV-1 p24 protein is not always present during advanced stages of HIV infection. To further confirm that these HIV proteins were from EVs, we tested the filtrate from ultracentrifugation (MW cutoff 100,000 kD) of HIV-positive urine, and no HIV proteins were present. We did not, however, perform an HIV infectivity assay, MAGI, on the isolated urinary EVs, and thus cannot be totally confident that HIV virions were not present in the EVs. HIV proteins in urinary EVs may be the result of a nonproductive HIV infection in the kidney [52–56] and/or EVs filtered from blood [21, 49, 57]. The type of HIV protein in the EVs remained relatively constant as demonstrated by the resampling of two patients, 203 and 311 days, after the first sample that had similar results. The identification of HIV proteins in urinary EVs may be a potential noninvasive diagnostic tool to monitor HIV disease states as well as treatment efficacy.

Different proteins and pathways were found in EVs from (1) CD4+ T cell > 300 versus <300 and (2) VLs < 200 versus >200 copies. It is interesting that EVs from HIV+ patients with low VLs and high CD4+ T cells, usually indicative of better health, had more proteins detected than EVs from high VLs and low CD4+ T cells (high VLs = 2486 vrs low = 15028; low CD4+ T cells = 2115 versus high CD4+ = 15761). These groups also had overlapping pathway results; however, proteins from high VLs and low CD4+ T cells did not have similar pathway results. Further comparison and analysis of the EV protein profile between the low VL/high CD4+ T cells and high VL/low CD4+ T cells may reveal more mechanisms involved in the evolving pathology of HIV infection.

Proteins contained in EVs can both enhance and inhibit host responses from innate, inflammatory, and adaptive reactions. Proteins from HIV+ patients showed a predominantly immunosuppressive profile. IL10 is a Th2 cytokine that downregulates macrophage function and inhibits T cell proliferation while IL6 can stimulate IL10 production and inhibit the effects of TNF-*α* and IL1. Both these cytokines were present in the EVs from HIV+ patients while TNF-*α* and IL1 were not detected suggesting an immunomodulatory effect may be elicited by the EVs. Other immune downregulating factors, IDO, HOXB4, HGF, and TGF*β*1, were found. IDO [58], HLA-G [59], and HGF [60] can inhibit natural killer cell activation which was one of the top biological processes found in the pathway analysis of the EV proteins in patients with high CD4+ T cells and low VLs. TGF*β*-1, an inhibitor of immune function, is induced by HIV Tat [61] and is a mediator of immune suppression in HIV infection [62–64]. These proteins were found in EVs from HIV+ patients while proinflammatory cytokines were not. New studies show that HIV+ nonprogressors have lower plasma TGF*β*-1 and IL10 than patients with progressive disease [65] and it is possible that EVs may sequester TGF*β*-1 and IL10 and remove them from circulation. The presence of over 16 different MHC Class I and II antigens in the EVs from HIV+ patients may support the hypothesis that this mechanism is used by intracellular pathogens to evade the immune response by decreasing cytotoxic T cell activity [66]. Herpes Simplex Virus-1 binds to HLA-DR inhibiting antigen presentation that leads to immune evasion [67]. Future studies should focus on the correlation of the concentration of these factors to HIV+ patients' clinical status.

In this study, we showed that structural, regulatory, and accessory HIV proteins could be detected in urinary EVs of HIV+ patients. Our WB analysis using polyclonal and monoclonal antibodies confirmed the presence of HIV proteins in the EVs from HIV+ patients. The most prevalent protein was HIV Nef. EVs from both* in vitro* and patient samples have been previously reviewed in [6]. HIV Nef induces an alternative pathway for TNF induction utilizing Notch-1, ADAM17, and Rab4+, all found in EVs from HIV+ patients, which leads to high plasma TNF levels [68]. Whether the isolation of these factors in EVs represents a diminishing or enhancement of TNF production remains to be examined.

The HIV Human Interaction database found significant interactions between HIV Nef, Vpr, Vif, and Vpu and human proteins. Serine/threonine protein kinases are important in T cell receptor signaling [69]. These kinases as well as CD4 and MHC antigens were found in EVs from the HIV+ samples; however, further studies are needed to determine the mechanisms involved with EV function in HIV infections. Cell adhesion molecules, ICAM, VCAM, and PECAM, were also found in the EVs from patients. Others have reported these molecules are present in HIV+ blood samples and may represent biomarkers from inflamed endothelium due to HIV infection [70].

One of the limitations of this study was a small sample size of specific HIV syndromes such as comorbidities, AIDS, HIV-associated nephropathy, and HIV-associated dementia as well as patients on or naïve to antiretroviral therapy. Increasing the numbers of HIV+ patients in these categories may allow us to determine whether specific HIV proteins as well as human proteins in urinary EVs could be associated with these conditions. Future studies will also quantify the amount of HIV proteins as well as human proteins to determine if a correlation exists between different HIV conditions and the amount of proteins detected.

HIV infection is usually detected by antibodies to HIV and can take up to three months to develop or by measuring VLs in blood whereas we can detect HIV-1 proteins in urinary EVs. In summary, urinary proteins in EVs from HIV+ patients may allow a noninvasive method to (1) rapidly screen for infection and identification of patients eligible for antiretroviral treatment (ART); (2) monitor ART treatment efficacy; and (3) diagnose HIV comorbidities.

## Figures and Tables

**Table 1:** Patient demographics.

Characteristics	HIV-positive (*N* = 35)	HIV-negative (*N* = 12)
Age (median ± IQR)	41.5 ± 14.25	59 ± 18
Sex (*n*, %)		
Male	25 (71.4%)	7 (58.3%)
Female	10 (28.6%)	5 (41.7%)
Race (*n*, %)		
African American/Black	28 (80%)	12 (100%)
White	7 (20%)	-
Hispanic	-	-
Asian	-	-
Viral loads (copies/ml) (median ± IQR)	50 ± 0	-
CD4+ T cell (cells/*µ*l) (median ± IQR)	66.5 ± 46.5	-
Antiretroviral therapy (*n*, %)	34 (97.1%)	-

**Table 2:** LC/MS/MS analysis of EV HIV proteins.

Accession	# AAs	MW [kDa]	Calc. pI	Description	Σ Coverage	Σ# peptides	Score A0	Coverage A0	# peptides A0
gi38491705	192	22.7	10.1	Vif protein [human immunodeficiency virus 1]	13.54	12	9.22	13.54	4
gi73913089	104	11.7	10.1	Gag protein [human immunodeficiency virus 1]	14.42	15	6.27	14.42	4
gi58374258	869	98.1	8.8	Envelope glycoprotein [human immunodeficiency virus 1]	1.5	5	5.63	1.5	3
gi183197180	404	45.8	8.4	Pol protein [human immunodeficiency virus 1]	3.47	3	5.27	3.47	3
gi255984636	160	18.1	5.3	Reverse transcriptase [human immunodeficiency virus 1]	7.5	4	4.80	7.5	2
gi256012108	114	13.5	5.7	Nef protein [human immunodeficiency virus 1]	14.04	3	4.53	14.04	2
gi9756252	524	60.4	8.7	Pol precursor [human immunodeficiency virus 1]	4.01	2	4.46	4.01	2
gi67082579	191	22.3	9.4	Reverse transcriptase [human immunodeficiency virus 1]	10.47	2	4.43	10.47	2
gi2290009	852	96.7	8.5	Envelope glycoprotein [human immunodeficiency virus 1]	7.16	11	4.33	5.87	3
gi167886806	25	2.7	8.7	Rev protein [human immunodeficiency virus 1]	56	4	4.29	56	2
gi23344577	99	10.6	9.4	Protease [human immunodeficiency virus 1]	12.12	5	4.36	12.12	2
gi4324808	1437	161.9	8.3	Gag-pol polyprotein [human immunodeficiency virus 1]	2.51	7	4.05	1.6	2
gi222533599	73	8.0	9.1	Env C2V3 protein [human immunodeficiency virus 1]	23.29	4	3.85	23.29	2
gi71060450	206	23.7	6.3	Negative factor [human immunodeficiency virus 1]	4.85	4	3.84	4.85	2
gi37935985	85	10.3	4.8	Vpu protein [human immunodeficiency virus 1]	11.76	4	3.84	11.76	2
gi108860432	870	98.8	8.5	gp160 [human immunodeficiency virus 1]	3.33	2	3.74	3.33	2
gi114801226	209	24.4	10.1	Tat protein [human immunodeficiency virus 1]	5.26	2	3.70	5.26	2
gi22596451	341	38.6	8.0	Truncated envelope glycoprotein [human immunodeficiency virus 1]	3.81	4	3.68	3.81	2
gi183200570	342	38.7	9.2	Truncated pol protein [human immunodeficiency virus 1]	3.51	2	3.68	3.51	2
gi34786230	176	19.8	9.6	gp120 protein [human immunodeficiency virus 1]	10.8	3	3.60	10.8	3
gi1002239	104	11.5	8.9	Envelope glycoprotein, v3 region [human immunodeficiency virus 1]	18.27	2	3.54	18.27	2
gi77168129	95	11.3	7.6	Vpr protein [human immunodeficiency virus 1]	13.68	3	3.40	13.68	2
gi222532593	129	14.7	8.4	Gag p17 protein [human immunodeficiency virus 1]	8.53	3	2.61	8.53	1
gi219688191	132	14.9	10.2	Matrix protein [human immunodeficiency virus 1]	9.09	6	2.56	9.09	1
gi255687141	288	32.1	7.7	Integrase [human immunodeficiency virus 1]	9.03	1	2.52	9.03	1
gi222532161	132	15.0	9.5	Gag p17 protein [human immunodeficiency virus 1]	9.09	6	2.45	9.09	1
gi37934078	573	65.0	9.0	Gag-pol fusion polyprotein [human immunodeficiency virus 1]	2.79	2	2.42	2.79	2
gi405003	207	23.1	7.7	gp120 [human immunodeficiency virus 1]	12.08	1	2.41	12.08	1
gi54792352	213	23.7	5.6	Gag polyprotein [human immunodeficiency virus 1]	9.39	2	2.34	9.39	2
gi3885826	132	14.9	9.6	p17 matrix [human immunodeficiency virus 1]	11.36	2	2.31	11.36	1

Accession # = NCBI NR database, #AAs = total number of amino acids in the protein entry, MW = molecular weight of the protein in kDa, Description: description from the NCBI database, and Peptides = total number of unique peptides found.

**Table 3:** Presence of HIV-1 proteins in HIV+ patient urinary EVs.

ID	ART	AIDS	Viral load	CD4 cells/ul	Nef	Gag	Pol	Protease	Rev	RT	Tat	Vif	p1	p24	p17	Poly	Vpu	Env	Vpr	Vif
			copies/ml																	
22	No			224	**X**	**X**	**X**	**X**	**X**		**X**	**X**	**X**	**X**	**X**					
27	Yes	AIDS	<50	134	**X**															
28	Yes	AIDS	280100	22	**X**	**X**		**X**		**X**		**X**								
30	Yes	AIDS	>10000	<20	**X**	**X**	**X**	**X**		**X**	**X**	**X**		**X**	**X**	**X**				
41	Yes		29187	440	**X**	**X**	**X**													
46	Yes		<50	689		**X**	**X**		**X**	**X**										
45	Yes		400	345	**X**			**X**	**X**									**X**		**X**
48	Yes		4974	454	**X**				**X**		**X**	**X**								
51			NA	NA	**X**	**X**	**X**	**X**		**X**										
52	Yes		51	574	**X**	**X**	**X**		**X**		**X**						**X**		**X**	**X**
61			<50	655		**X**	**X**													
62	Yes	AIDS	<50	232	**X**															
63	No		2023	83	**X**															
65	No		NA	NA														**X**		
66	No		NA	NA			**X**													
67	Yes		75	509	**X**															
68	No		NA	NA														**X**		
69	Yes		<50	187	**X**															
70	Yes		<50	399	**X**															
71	Yes		<50	456														**X**		
74			NA	NA														**X**		
86	Yes		<75	1642	**X**	**X**	**X**	**X**												
103	Yes	AIDS	150	560	**X**	**X**	**X**													
104	Yes	AIDS	77	313	**X**	**X**														
108	Yes	AIDS	<50	653	**X**	**X**	**X**													
110	Yes		<50	379	**X**	**X**														
111	Yes	AIDS	<50	182		**X**	**X**													
112	Yes	AIDS	>200	581			**X**			**X**										
142	Yes		<50	487	**X**	**X**	**X**		**X**		**X**					**X**	**X**			**X**
173-1	Yes		<50	398	**X**	**X**	**X**	**X**	**X**	**X**	**X**	**X**		**X**			**X**			
173-2	Yes		<50	398	**X**	**X**	**X**	**X**		**X**		**X**								
174-1	Yes		48	315	**X**	**X**	**X**		**X**	**X**		**X**								
174-2	Yes		48	315	**X**	**X**	**X**					**X**								
196-1	Yes	AIDS	<50	113	**X**	**X**	**X**	**X**	**X**	**X**	**X**	**X**		**X**	**X**	**X**	**X**			**X**
196-2	Yes	AIDS	<50	113	**X**	**X**	**X**	**X**	**X**	**X**	**X**	**X**		**X**	**X**	**X**	**X**			**X**

An initial protein identification list was generated from matches with an Xcorr score versus charge state of 1.0 (+1), 1.5 (+2), and 1.7 (+3) and consensus scores greater than 10.0; NA = not available.

**Table 4:** Exosomal proteins found in urinary EVs from HIV+ patients.

	Genes in our analysis	Genes in the FunRich database	Percentage of genes	Fold enrichment
Exosomal proteins	1932	2001	29.44	2.11

A1BG, *A2M*, AARS, ABCA7, ABCB1, ABCB11, ABCB6, ABCC1, ABCC11, ABCC9, ABCG2, ABHD8, ACAA2, ACAT1, ACAT2, ACE, ACE2, *ACLY*, ACO1, ACOT11, ACP2, ACSL3, ACSL4, ACSM1, **ACTA1**, **ACTA2**, ***ACTB***, **ACTBL2**, **ACTC1**, ***ACTG1***, **ACTL6A**, **ACTN1**, **ACTN2**, ***ACTN4***, **ACTR1A**, **ACTR1B**, **ACTR2**, **ACTR3**, ACY1, ACY3, ADAM10, ADAMTS3, ADCY1, ADH5, ADH6, ADK, ADSL, AEBP1, AGAP2, AGR2, AGR3, AGRN, AGT, AHCTF1, AHCYL1, AHNAK, AHSA1, AHSG, AK1, AK2, AKAP9, AKR1A1, AKR1B10, ALAD, *ALB*, ALCAM, ALDH16A1, ALDH1A1, ALDH1L1, ALDH2, ALDH3B1, ALDH8A1, *ALDOA*, ALDOB, ALDOC, ALK, ALOX12, ALPL, ALPP, ALYREF, AMBP, AMN, ANGPT1, ANGPTL1, ANGPTL4, ANKFY1, ANKRD11, ANO1, ANO6, *ANPEP*, *ANXA1*, *ANXA11*, ANXA13, ANXA3, *ANXA4*, *ANXA6*, ANXA7, AOX1, AP1M1, AP2A1, AP2A2, AP2M1, AP4M1, APAF1, APLP2, APOA1, APOA2, APOB, APOD, APOE, APOL1, APP, APPL1, APPL2, APRT, AQP2, ARF5, ARFIP1, ARHGAP1, ARHGAP23, ARHGDIA, ARHGDIB, ARHGEF12, ARHGEF18, ARL15, ARL3, ARL8B, ARMC3, ARMC9, ARPC1A, ARPC1B, ARPC2, ARPC3, ARPC5, ARRDC1, ARSE, ARSF, ARVCF, ASAH1, ASB6, ASL, ASNA1, ASNS, ATAD2, ATIC, *ATP1A1*, ATP1A2, ATP1A3, ATP2B1, ATP2B2, ATP2B4, ATP4A, ATP5A1, ATP5B, ATP5L, ATP6AP1, ATP6AP2, ATP6V0A1, ATP6V0A4, ATP6V0C, ATP6V0D1, ATP6V0D2, ATP6V1A, ATP6V1B1, ATP6V1C1, ATP6V1C2, ATP6V1D, ATP6V1E1, ATP6V1H, ATRN, AUP1, AZGP1, AZU1, B2M, B3GAT3, B4GALT1, B4GALT3, BAIAP2, BAIAP2L1, BASP1, BAZ1B, BCAM, BCR, BDH2, BGN, BHLHB9, BHMT, BHMT2, BLMH, BLOC1S5, BLVRA, BLVRB, BMP3, BPI, BPIFB1, BPTF, BRI3BP, BROX, *BSG*, BTG2, BTN1A1, C11orf52, C11orf54, C16orf80, C16orf89, C17orf80, C19orf18, C1GALT1C1, C1orf116, C1QC, C1QTNF1, C1QTNF3, C1R, C2orf16, C3, C4BPA, C5, C9, CAB39L, CACNA2D1, CACYBP, CAD, CALM1, CALML3, CALR, CAMK4, CAMP, CAND1, CANX, CAP1, CAPN1, CAPN2, CAPN5, CAPN7, CAPNS1, CAPS, CAPZA2, CAPZB, CARD11, CASP9, CAV1, CBR3, CC2D1A, CCDC105, CCDC132, CCL28, CCPG1, *CCT2*, *CCT3*, CCT4, *CCT5*, CCT6A, CCT7, CCT8, CD101, CD14, CD163L1, ***CD19***, CD2, CD22, CD274, CD2AP, CD300A, CD36, ***CD37***, CD40, CD44, CD53, CD55, CD58, CD59, ***CD63***, CD70, CD74, CD79B, CD80, ***CD81***, ***CD9***, CD97, *CDC42*, CDC42BPA, CDC42BPB, CDH1, CDH17, CDHR2, CDHR5, CDK1, CDK5RAP2, CDKL1, CEACAM5, CELSR2, CEMIP, CEP250, CES2, CETP, CFD, CFH, CFI, *CFL1*, CHGB, CHID1, CHMP1A, CHMP2B, CHMP4B, CHRDL2, CHST1, CHST14, CIB1, CKAP4, CKB, CLASP1, CLCA4, CLDN3, CLDN4, CLDN7, CLIC1, CLIC4, CLIC5, CLIC6, CLIP2, CLSTN1, *CLTC*, CLTCL1, CLU, CMPK1, CNDP2, CNKSR2, CNTLN, COASY, COBLL1, COL12A1, COL15A1, COL18A1, COL6A1, COL6A2, COL6A3, COLEC10, COLGALT1, COMT, COPA, COPB1, COPB2, COPS8, CORO1A, CORO1B, COX4I1, COX5B, CP, CPD, CPN2, CPNE1, CPNE3, CPNE5, CPNE8, CPVL, CR1, CR2, CRB2, CREB5, CRISPLD1, CRNN, CRTC2, CRYAB, CRYZ, CS, CSE1L, CSK, CSPG4, CSRP1, CST4, CSTB, CTDSPL, CTNNA1, CTNNB1, CTNND1, CTSB, CTSC, CTSG, CTTN, CUBN, CUL3, CUL4B, CUTA, CUX2, CXCR4, CYB5R1, CYBRD1, CYFIP1, CYFIP2, CYP2J2, DAAM2, DAG1, DAK, DARS, DBNL, DCD, DCTN2, DCXR, DDAH1, DDAH2, DDB1, DDC, DDR1, DDX11, DDX19A, DDX19B, DDX21, DDX23, DDX3X, DDX5, DERA, DHCR7, DHX34, DHX36, DHX9, DIAPH2, DIP2A, DIP2B, DIP2C, DLD, DLG1, DMBT1, DNAH7, DNAH8, DNAJA1, DNAJA2, DNAJB1, DNAJB9, DNAJC13, DNAJC3, DNAJC7, DNHD1, DNM2, DNPH1, DOCK10, DOCK2, DOPEY2, DPEP1, DPP3, DPP4, DPYS, DPYSL2, DSC2, DSG2, DSG3, DSP, DSTN, DUOX2, DUSP26, DUT, DYNC1H1, DYNC2H1, DYSF, ECE1, ECH1, ECM1, EDIL3, EEA1, *EEF1A1*, EEF1A2, EEF1D, EEF1G, *EEF2*, EFEMP1, EFEMP2, EGF, **EGFR**, EHD1, EHD2, EHD3, *EHD4*, EIF2S1, EIF2S3, EIF3A, EIF3B, EIF3E, EIF3L, EIF4A1, EIF4A2, EIF4A3, EIF4E, EIF4G1, EIF4H, ELANE, EML5, *ENO1*, ENO2, ENO3, ENPP3, ENPP4, ENPP6, ENTPD1, EPB41L2, **EPCAM**, EPHA2, EPHA5, EPHB1, EPHB2, EPHB3, EPHB4, EPHX2, EPN3, EPPK1, EPRS, EPS8, EPS8L1, EPS8L2, EPS8L3, ERAP1, ERBB2, ERMN, ERO1L, ERP44, ESD, ETFA, EVPL, EXOC4, EXOSC10, EXT2, EYS, *EZR*, F11, F11R, F5, F7, FABP1, FABP3, FAH, FAM129A, FAM129B, FAM151A, FAM208B, FAM209A, FAM20A, FAM20C, FAM49B, FAM65A, FAS, FASLG, *FASN*, FAT1, FAT2, FBL, FBP1, FBP2, FCGBP, FCN1, FCN2, FERMT3, FGA, FGB, FGG, FGL2, FGR, FH, FIGNL1, FKBP1A, FKBP4, FKBP5, *FLNA*, FLNB, FLNC, **FLOT1**, FLOT2, FMNL1, FN1, FOLH1, FRK, FSCN1, FTCD, FUCA1, FURIN, FUS, FUT2, FUT3, FUT6, FUT8, FUZ, G6PD, GAA, GABRB2, GAL3ST4, GALK1, GALM, GALNT3, GANAB, GARS, GART, GATSL3, GBE1, GBP6, GCN1L1, GCNT2, GCNT3, GDF2, *GDI2*, GDPD3, GEMIN4, GFPT1, GGCT, GGH, GGT1, GHITM, GIPC1, GK, GK2, GLB1, GLDC, GLG1, GLIPR2, GLO1, GLUD1, GLUL, GNA13, GNAI1, *GNAI2*, GNAQ, *GNAS*, *GNB1*, *GNB2*, GNB2L1, GNB3, GNB4, GNB5, GNG12, GNPDA1, GNPTG, GOLGA4, GOLGA7, GOT1, GOT2, GPC1, GPC4, GPD1, GPI, GPM6A, GPR155, GPR64, GPR98, GPRASP1, GPRC5A, GPRC5B, GPT, GREB1, GRHPR, GRID1, GRIN1, GRK4, GSN, GSR, GSS, GSTA3, GSTCD, GSTK1, GSTO1, GSTP1, GUSB, H1FOO, H2AFY, H2AFY2, HADHA, HAPLN3, HAUS5, HBB, HBD, HBS1L, HDHD2, HEBP1, HEBP2, HEPH, HGD, HGS, HINT1, HIRA, HIST1H1B, HIST1H2BA, HIST1H2BL, HIST2H2AC, **HLA-A**, **HLA-B**, HLA-DPB1, HLA-DQB1, **HLA-DRB1**, **HLA-DRB5**, HLAE, HNMT, HNRNPA1, HNRNPA2B1, HNRNPC, HNRNPF, HNRNPK, HNRNPL, HP, HPD, HPGD, HPR, HPRT1, HRG, HRNR, HSD17B10, HSD17B4, ***HSP90AB1***, ***HSP90B1***, HSPA12A, HSPA13, HSPA1L, HSPA2, HSPA4, *HSPA5*, HSPA6, *HSPA8*, HSPA9, HSPB1, HSPB8, HSPD1, HSPG2, HSPH1, HTATIP2, HTRA1, HUWE1, HYOU1, IARS, **ICAM1**, ICAM3, IDH1, IFITM2, IFITM3, IGF2R, IGFALS, IGSF3, IGSF8, IKZF5, IMPDH2, INADL, INSR, IQCB1, IQCG, IQGAP1, IQGAP2, IRF6, IST1, ITFG3, ITGA1, ITGA2, ITGA2B, ITGA3, **ITGA4**, *ITGA6*, ITGAL, ITGAV, *ITGB1*, ITGB2, ITGB3, ITGB4, ITGB7, ITGB8, ITIH2, ITIH4, ITM2C, ITSN1, ITSN2, IVL, JADE2, JUP, KALRN, KCNG2, KHK, KIAA1324, KIF12, KIF15, KIF18B, KIF3A, KIF3B, KIF9, KIFC3, KL, KNG1, *KPNB1*, KPRP, KRT1, KRT10, KRT12, KRT14, KRT15, KRT16, KRT17, KRT18, KRT19, KRT2, KRT20, KRT24, KRT25, KRT27, KRT28, KRT3, KRT5, KRT6C, KRT7, KRT73, KRT75, KRT76, KRT77, KRT78, KRT79, KRT8, KRT9, ***L1CAM***, LAD1, LAMA3, LAMA4, LAMA5, LAMB2, LAMB3, LAMC1, LAMC2, LAMP1, ***LAMP2***, LAMTOR3, LBP, LCK, LCP1, *LDHA*, LDHB, LEPRE1, LFNG, LGALS3, *LGALS3BP*, LGALS4, LIMA1, LIN7A, LIN7C, LMAN1, LMAN2, LOXL4, LPO, LRP1, LRP1B, LRP2, LRP4, LRPPRC, LRRC15, LRRC16A, LRRC57, LRRK2, LRSAM1, LSP1, LSR, LTA4H, LTBP3, LTF, LUZP1, LYPLA2, MAGI3, MAL2, MAN1A1, MAN1A2, MAN2A1, MAP4K4, MAP7, MARCKSL1, MARK3, MARS, MARVELD2, MASP1, MASP2, MBD5, MBLAC2, **MCAM**, MCPH1, MDH1, MDH2, MEGF8, MEP1A, MEST, METRNL, *MFGE8*, MFI2, MGAM, MGAT1, MGAT4A, MID2, MIF, MINK1, MLLT3, MLLT4, MME, MMP24, MMP25, MMRN1, MMRN2, MNDA, MOB1A, MOB1B, MOGS, MPO, MPP5, MPP6, MS4A1, MSH6, *MSN*, MSRA, MTA1, MTAP, MTCH2, MTHFD1, MTMR11, MTMR2, MUC13, MUC16, MUC4, MUM1L1, MVB12A, MVB12B, *MVP*, MX1, MXRA5, MXRA8, MYADM, MYH10, MYH11, MYH13, MYH14, MYH3, MYH8, *MYH9*, MYL6B, MYO15A, MYO1B, MYO1C, MYO1D, MYO1E, MYO1G, MYO5B, MYO6, MYOF, N4BP2L2, NAA16, NAA50, NACA, NAGLU, NAMPT, NAP1L4, NAPA, NAPG, NAPRT, NAPSA, NARS, NBR1, NCALD, NCCRP1, NCKAP1, NCKAP1L, NCL, NCOA3, NCSTN, NDRG1, NDRG2, NEB, NEBL, NEDD4, NEDD4L, NEDD8, NEU1, NID1, NIN, NIPBL, NIT2, NKX61, NONO, NOTCH1, NOX3, NPC1, NPEPPS, NPHS1, NPHS2, **NPM1**, NPNT, NQO2, NT5C, NT5E, NUCB1, NUCB2, NUDT5, NUMA1, NXPE4, OLA1, OPTN, OR2A4, OS9, OSBPL1A, OXSR1, P2RX4, P4HB, PA2G4, PACSIN2, PACSIN3, PADI2, PAFAH1B1, PAFAH1B2, PAGE2, PAICS, PAM, PARD6B, PARP4, PBLD, PCBP1, PCDHGB5, PCK1, PCLO, PCNA, PCSK9, PCYOX1, PDCD2, PDCD5, PDCD6, ***PDCD6IP***, PDDC1, PDE8A, PDIA2, PDIA3, PDIA4, PDIA6, PDLIM7, PDZK1, PEBP1, PECAM1, PEF1, PEPD, PEX1, PFAS, PFKL, PFKP, PGAM1, PGD, *PGK1*, PGLYRP1, PGM1, PHB2, PHGDH, PI4KA, PIGR, PIK3C2A, PIK3C2B, PILRA, PIP, PIP4K2C, PKD1, PKD1L3, PKD2, PKHD1, PKLR, *PKM*, PKN2, PKP3, PLAT, PLAU, PLCB1, PLCD1, PLCG2, PLD3, PLEC, PLEKHA1, PLEKHA7, PLEKHB2, PLG, PLIN2, PLOD1, PLOD2, PLOD3, PLS1, PLSCR1, PLTP, PLVAP, PLXNA1, PLXNB2, PM20D1, PMEL, PNP, PODXL, POFUT2, PON1, PON3, POTEE, POTEF, POTEI, POTEM, PPA1, PPARG, PPFIA2, *PPIA*, PPIB, PPL, PPM1L, PPP1CB, PPP1R7, PPP2CA, PPP2R1A, PPP2R1B, PRCP, *PRDX1*, PRDX3, PRDX4, PRDX5, PRG4, PRKAR2A, PRKCA, PRKCD, PRKCH, PRKCI, PRKCZ, PRKDC, PRKRIP1, PRNP, PROM1, PROM2, PROS1, PROZ, PRRC2A, PRSS23, PRTN3, PSAP, PSAT1, PSMA2, PSMA3, PSMA5, PSMA7, PSMB1, PSMB3, PSMB4, PSMB5, PSMB6, PSMB8, PSMB9, PSMC2, PSMC4, PSMC6, PSMD11, PSMD12, PSMD13, PSMD2, PSME1, PSME2, PSME3, PTBP1, PTER, *PTGFRN*, PTGR1, PTGS1, PTPN13, PTPN23, PTPRA, **PTPRC**, PTPRF, PTPRJ, PTPRO, PTRF, PTX3, PYGB, PYGL, QDPR, QPCT, QPRT, QSOX1, RAB10, RAB11B, RAB17, *RAB1A*, RAB1B, RAB22A, RAB25, RAB29, RAB2A, RAB34, RAB3B, RAB3GAP1, RAB43, RAB4B, RAB6B, *RAB7A*, *RAB8A*, RAB8B, RAB9A, *RAC1*, RACGAP1, RALA, RALB, RAP1A, *RAP1B*, RAP1GDS1, RAP2A, RAPGEF3, RARRES1, RARS, RASAL3, RASSF9, RBL2, RCC2, REG4, RELN, RENBP, RFC1, RFTN1, RHEB, RHOB, RHOF, RIMS2, RLF, RNASE7, RNF213, RNH1, RNPEP, ROBO2, ROCK2, RP2, RPL10, RPL10A, RPL14, RPL15, RPL23, RPL3, RPL30, RPL34, RPL35A, RPL4, RPL5, RPL6, RPL8, RPLP2, RPN1, RPS11, RPS14, RPS15A, RPS16, RPS18, RPS2, RPS20, RPS21, RPS27A, RPS3A, RPS4X, RPS4Y1, RPS4Y2, RPS7, RPS9, RRAS, RREB1, RSU1, RTN4, RUSC2, RUVBL1, RUVBL2, RYR1, S100A11, S100A6, S100P, SAA1, SAFB2, SAMM50, SARS, SBSN, SCAMP2, SCAMP3, SCARB1, SCARB2, SCEL, SCIN, SCN10A, SCN11A, SCPEP1, SCRIB, SCRN2, *SDCBP*, SDF4, SEC31A, SELENBP1, **SELP**, SEMA3G, SEPP1, SERBP1, SERINC1, SERINC2, SERINC5, SERPINA1, SERPINA3, SERPINA4, SERPINA5, SERPINA7, SERPINB1, SERPINB13, SERPINB6, SERPINB9, SERPING1, SETD4, SFI1, SFN, SFRP1, SFT2D2, SH3BP4, SHMT1, SHMT2, SHROOM2, SIAE, SIRPA, SIT1, SLAMF1, SLAMF6, SLC12A1, SLC12A2, SLC12A3, SLC12A7, SLC12A9, SLC13A2, SLC13A3, SLC15A2, *SLC16A1*, SLC1A1, SLC1A4, SLC1A5, SLC20A2, SLC22A11, SLC22A12, SLC22A13, SLC22A2, SLC22A5, SLC22A6, SLC23A1, SLC25A1, SLC25A3, SLC25A4, SLC25A6, SLC26A11, SLC26A4, SLC26A9, SLC27A2, SLC29A1, SLC2A1, SLC2A3, SLC34A2, SLC35D1, SLC36A2, SLC37A2, SLC38A1, SLC39A5, SLC3A1, *SLC3A2*, SLC44A1, SLC44A2, SLC44A4, SLC46A3, SLC4A1, SLC4A4, SLC5A1, SLC5A10, SLC5A2, SLC5A5, SLC5A6, SLC5A8, SLC5A9, SLC6A13, SLC6A14, SLC6A19, SLC7A5, SLC9A1, SLC9A3, SLC9A3R1, SLC9A3R2, SLCO4C1, SLIT2, SLK, SMC2, SMC3, SMIM22, SMIM24, SMO, SMPDL3B, SMURF1, SNCG, SND1, SNRNP200, SNX12, SNX18, SNX25, SNX33, SNX9, SOD1, SOGA1, SORD, SORL1, SORT1, SPAG9, SPAST, SPEN, SPINK1, SPON2, SPRR3, SPTAN1, SPTBN1, SQSTM1, SRC, SRPR, SRSF7, ST13, ST3GAL1, ST3GAL6, STAMBP, STAU1, STIP1, STK10, STK11, STK24, *STOM*, STRIP1, STX3, STX4, STX7, STXBP1, STXBP2, STXBP3, STXBP4, SUB1, SUCLA2, SUSD2, SYAP1, SYNE1, SYNE2, TAB3, TACSTD2, TAF6L, TALDO1, TAOK1, TARS, TAX1BP1, TAX1BP3, TBC1D10A, TBC1D21, TC2N, *TCP1*, TECTA, TEKT3, TEX14, **TF**, *TFRC*, TGFB1, TGFBI, TGFBR3, TGM1, TGM2, TGM3, TGM4, *THBS1*, THBS2, THRAP3, THSD4, THY1, TIAM2, TINAGL1, TJP2, *TKT*, TLN1, TLR2, TM7SF3, TM9SF2, TMBIM1, TMC6, TMC8, TMED2, TMED9, TMEM109, TMEM192, TMEM2, TMEM256, TMEM27, TMEM63A, TMPRSS11B, TMPRSS11D, TMPRSS2, TNFAIP3, TNFRSF8, TNFSF10, TNFSF13, TNIK, TNKS1BP1, TNPO3, TOLLIP, TOM1, TOM1L2, TOMM70A, TOR1A, TOR1B, TOR3A, *TPI1*, TPM3, TPP1, TPRG1L, TRAP1, TREH, TRIP10, TSNAXIP1, TSPAN1, TSPAN15, TSPAN3, TSSK3, TSTA3, TTC17, TTC18, TTLL3, TTN, TTR, *TUBA1B*, TUBA4A, TUBB3, TUBB4A, TUBB8, TUFM, TWF2, TXNDC16, TXNDC8, TXNRD1, TYK2, TYRP1, UACA, *UBA1*, UBAC1, UBASH3A, UBE2N, UBE2V2, UBL3, UBXN6, UEVLD, UGDH, UGGT1, UGP2, ULK3, UMOD, UPB1, UPK1A, UPK3A, UQCRC2, UTRN, UXS1, VAMP1, VAMP3, VAMP7, VAPA, VASN, VASP, VAT1, VCL, *VCP*, VDAC3, VIL1, VIM, VMO1, VPS13C, VPS13D, VPS28, VPS35, VPS36, VPS37B, VPS37C, VPS37D, VPS4A, VPS4B, VTA1, VWA2, VWF, WARS, WAS, WASF2, WASL, WDR1, WIZ, WNT5B, XDH, XPNPEP2, XPO1, XRCC5, XRCC6, YBX1, YES1, *YWHAE*, *YWHAG*, *YWHAH*, *YWHAZ*, ZCCHC11, ZDHHC1, ZFYVE20, ZG16B, ZMPSTE24, ZNF114, ZNF486, ZNF571, and ZNHIT6.				

*GENE*: ExoCarta (http://exocarta.org/exosome_markers_new) [33]; **GENE**: EV antibody array [35]; GENE: HIV exosomal proteins [36].

**Table 5:** Exosomal proteins found in urinary EVs from uninfected controls.

	Genes in our analysis	Genes in the FunRich database	Percentage of genes	Fold enrichment
Exosomal proteins	37	2001	72.54	5.26

A1BG, ACTA1, ACTA2, ACTB, ACTBL2, ACTC1, ACTG1, ACTG2, ALB, AMBP, APOA1, APOD, AZGP1, B2M, CDH1, CLU, CP, CRNN, DCTN2, EGF, HP, HPR, HSPB1, ITIH4, KNG1, LAMA3, LMAN2, POTEE, POTEF, POTEI, S100A8, SERPINA1, SERPING1, TF, TTR, UMOD, and VASN.

**Table 6:** Functional enrichment analysis of HIV+ EV proteins.

	Genes in the dataset	Genes in the Bkg. database	Percentage of genes	Fold enrichment	Corrected *P* value (BH FDR)
*Molecular function*					
Protein serine/threonine kinase activity	272	5,602	30	1.18	1.04^−08^
Catalytic activity	456	827	4.9	1.1	1.12^−05^
GTPase activator activity	131	836	4.7	1.2	8.14^−05^
Guanyl-nucleoside exchange factor activity	105	614	3.6	1.2	8.54^−05^
Cell adhesion molecule activity	307	531	3.3	1.1	0.0001
*Biological process*					
Regulation of nucleobase, nucleoside, and nucleic acid	2,236	4,658	24.8	1.05	3.24^−05^
*Biological pathway*					
Integrin cell surface interactions	69	1,366	23.3	1.2	0.03

**Table 7:** Comparison of pathways between HIV+ groups from Pathway Studio 11.4.

HIV group	Pathway
CD4+ T cells greater than 300 *n* = 15	Natural killer cell inhibitor receptor signaling Intermediate filament polymerization Ca2+ flux regulation G1/S phase transition G2/M phase transition S/G2 phase transition Protein folding Golgi to endosome transport Endosomal recycling Kinetochore assembly

CD4+ T cells less than 300 *n* = 15	Neutrophil chemotaxis Vascular motility Platelet activation via GPCR signaling Insulin influence on protein synthesis mTOR signaling overview EDNRA/B → vascular motility Proplatelet maturation Natural killer cell activation through ITAM-containing receptors Taste sensor receptors activates mTOR signaling Natural killer cell activation

Low VLs *n* = 14	Intermediate filament polymerization Natural killer cell inhibitory receptor signaling golgi to endosome transport Ca+ flux regulation HRH1/3 → synaptic transmission Vascular motility Endosomal recycling G1/S phase transition Golgi transport G2/M phase transition

High VLs *n* = 10	Metaphase/anaphase phase transition S/G2 phase transition Spindle assembly Natural killer cell activation Histone ubiquitylation Eosinophil survival by cytokine signaling Protein folding G2/M phase transition

**Table 8:** Functional enrichment analysis of control EV proteins.

	Genes in the database	Genes in the Bkg. database	Percentage of genes	Fold enrichment	Corrected *P* value (BH FDR )
*Site of expression*					
Cervicovaginal fluid	16	544	12.0	4.2	2.59*E* − 06
Neutrophils	13	392	9.7	4.8	6.68*E* − 06
Gastric juice	9	222	6.7	6.1	4*E* − 05
*Molecular function*					
Defense/immunity protein activity	5	52	3.7	15.7	3.96*E* − 05
*Biological process*					
Immune response	13	561	9.8	3.4	0.00026
Signal transduction	43	3907	32.5	1.5	0.0026
Cell communication	41	3687	31.1	1.5	0.0028
Antigen presentation	1	1	0.7	134.4	0.0073

**Table 9:** Overlapping EV proteins from HIV+ and HIV− samples, LC/MS/MS analysis.

Gene	
ABCB1	ATP-binding cassette, subfamily B (MDR/TAP), member 1
ANXA8	Annexin A8
ASIC1	Acid-sensing (proton-gated) ion channel 1
ASIC2	Acid-sensing (proton-gated) ion channel 2
AUTS2	Autism susceptibility candidate 2
AZU1	Azurocidin 1
BCAT1	Branched chain amino acid transaminase 1, cytosolic
BRD4	Bromodomain containing 4
CCL5	Chemokine (C-C motif) ligand 5
CEACAM8	Carcinoembryonic antigen-related cell adhesion molecule 8
CFH	Complement factor H
CHIT1	Chitinase 1 (chitotriosidase)
CLDN7	Claudin 7
COL16A1	Collagen, type XVI, alpha 1
CPB2	Carboxypeptidase B2 (plasma)
CRADD	CASP2 and RIPK1 domain containing adaptor with death domain
CTSG	Cathepsin G
CYP4A11	Cytochrome P450, family 4, subfamily A, polypeptide 11
DEFA1	Defensin, alpha 1
DNAH17	Dynein, axonemal, heavy chain 17
DUSP9	Dual specificity phosphatase 9
EIF4A1	Eukaryotic translation initiation factor 4A1
ELANE	Elastase, neutrophil expressed
	v-erb-b2 avian erythroblastic leukemia viral oncogene homolog 2
FARP1	FERM, RhoGEF (ARHGEF), and pleckstrin domain protein 1 (chondrocyte-derived)
GDF15	Growth differentiation factor 15
GNA12	Guanine nucleotide binding protein (G protein) alpha 12
GNL1	Guanine nucleotide binding protein-like 1
GRIN2A	Glutamate receptor, ionotropic, N-methyl D-aspartate 2A
HAAO	3-Hydroxyanthranilate 3,4-dioxygenase
HAL	Histidine ammonia-lyase
HBA1	Hemoglobin, alpha 1
HBB	Hemoglobin, beta
HBD	Hemoglobin, delta
IGKC	Immunoglobulin kappa constant
LGALS3	Lectin, galactoside-binding, soluble, 3
MEF2C	Myocyte enhancer factor 2C
MLLT4	Myeloid/lymphoid or mixed-lineage leukemia (trithorax homolog, Drosophila); translocated to, 4
MPO	Myeloperoxidase
MRC2	Mannose receptor, C type 2
MYBPC3	Myosin binding protein C, cardiac
NCAM1	Neural cell adhesion molecule 1
NKTR	Natural killer-tumor recognition sequence
NUP93	Nucleoporin 93 kDa
PDE1C	Phosphodiesterase 1C, calmodulin-dependent 70 kDa
PDLIM5	PDZ and LIM domain 5
PIK3R1	Phosphoinositide-3-kinase, regulatory subunit 1 (alpha)
RAB31	RAB31, member RAS oncogene family
RAP1GAP	RAP1 GTPase activating protein
REG1A	Regenerating islet-derived 1 alpha
RNASE2	Ribonuclease, RNase A family, 2 (liver, eosinophil-derived neurotoxin)
RNASE3	Ribonuclease, RNase A family, 3
RPS14	Ribosomal protein S14
RUNX2	Runt-related transcription factor 2
SHBG	Sex hormone-binding globulin
SLC22A5	Solute carrier family 22 (organic cation/carnitine transporter), member 5
SLC6A6	Solute carrier family 6 (neurotransmitter transporter), member 6
TACC2	Transforming, acidic coiled-coil containing protein 2
TAF6L	TAF6-like RNA polymerase II, p300/CBP-associated factor (PCAF)-associated factor, 65 kDa
TNIK	TRAF2 and NCK interacting kinase
TRAPPC12	Trafficking protein particle complex 12
TRIM58	Tripartite motif containing 58
WNT2B	Wingless-type MMTV integration site family, member 2B
WNT6	Wingless-type MMTV integration site family, member 6

**Table 10:** Functional analysis of overlapping HIV+ and HIV− EV proteins.

	Genes in the data set	Genes in the Bkg. database	Percentage of genes	Fold enrichment	Corrected *P* value (Bonferroni method)
*Site of expression*					
Urine	31	3202	51.7	3.0	6.85*E* − 07
Cervicovaginal fluid	12	544	20.0	7.2	5.33*E* − 05
Neutrophils	9	392	15.0	7.7	1.56*E* − 03
032403_BALF4_glypep	4	43	6.7	35.0	3.53*E* − 03
Neutrophil	19	1979	31.7	3.0	3.67*E* − 03
Monocyte	23	2786	38.3	2.6	3.90*E* − 03
*Cellular component*					
Extracellular	22	1808	37.9	3.1	4.61*E* − 05
Stored secretory granule	3	19	5.2	51.0	3.27*E* − 03
Lysosome	17	1609	29.3	2.8	7.73*E* − 03
Extracellular space	8	399	13.8	5.6	1.05*E* − 02
Exosomes	19	2001	32.8	2.5	1.14*E* − 02
Azurophil granule	2	6	3.4	108.9	1.35*E* − 02

## Supplementary Materials

Supplementary 1 Top 10 biological function pathways using Pathway Studio 11 Mammal Plus, Elsevier, Inc., for HIV+ EV proteins from HIV+ patients with (1) CD4+ T cells greater than 300, (2) CD4+ T cells less than 300, (3) viral loads less than 200 copies, and (4) viral loads greater than 200 copies.

Supplementary 2 EV HIV protein interactions (Nef, Vif, Vpr, and Vpu) with human proteins identified using HIV-1 Human Interaction database (https://www.ncbi.nlm.nih.gov/genome/viruses/retroviruses/hiv-1/interactions/). The file includes the gene symbol, human protein name, interaction keywords, protein accession ID, and PMID of references citing the interaction.
