# Supplementary material for: Detection of HIV-1 and Human Proteins in Urinary Extracellular Vesicles from HIV+ Patients
Source: Adv Virol. 2018 Mar 12;2018:7863412. doi: 10.1155/2018/7863412 (PMC5867598; doi:10.1155/2018/7863412)
Supplement: Supplementary 1 — Top 10 biological function pathways using Pathway Studio 11 Mammal Plus, Elsevier, Inc., for HIV+ EV proteins from HIV+ patients with (1) CD4+ T cells greater than 300, (2) CD4+ T cells less than 300, (3) viral loads less than 200 copies, and (4) viral loads greater than 200 copies. [file 7863412.f1.pdf]

PATHWAY STUDIO

Entity List Summary Analysis for CD4+ T cells greater than 300

generated by **Pathway Studio**

Summary

Group: CD4 greater than 300

# of Entities: 15761

This summary report contains 1 result with 10 pathways.

Table of content

Top Pathways

- Biological Function
  - Natural Killer Cell Inhibitory Receptor Signaling
  - Intermediate Filament Polymerization
  - Ca2+ Flux Regulation
  - G1/S Phase Transition
  - G2/M Phase Transition
  - S/G2 Phase Transition
  - Protein Folding
  - Golgi to Endosome Transport
  - Endosomal Recycling
  - Kinetochore Assembly

Top Pathways

Biological Function

Name: Pathways/Groups similar to CD4 greater than 300

Name	Parent Folder	# of Entities	Expanded # of Entities	Overlap	Percent Overlap	Overlapping Entities	p-value	Jaccard similarity
**Natural Killer Cell Inhibitory Receptor Signaling**	Lymphatic System; Natural Killer Cell Receptors Signaling; Natural Killer Cell Receptors Signaling (Immunological Pathways)	62	109	85	77	LILRB1, CEACAM1, ABL1, VAV3, BAIAP2, CD300A, CDH9, CDH5, CDH6, CDH1, CDH2, CDH8, CDH4, CDH7, CDH3, CDH10, CDH11, CDH16, CDH18, CDH15, CDH17, CDH13, CDH12, CBL, DOK1, COL1A1, COL1A2, CRK, CSK, PCDHGA12, PCDHB5, FAT1, FAT2, SIGLEC9, RAPGEF1, CDH20, HLA-A, HLA-B, FYN, LAT, SIGLEC7, INPP5D, HLA-C, HLA-E, HLA-G, CDH19, KIR2DL1, KIR3DL2, CLEC2D, KLRC1, KLRB1, LAIR1, LCK, LCP2, LYN, PAK1, PCDH8, TIGIT, MAP2K1, PCDH1, PCDH7, PCDHGC3, PCDH9, DCHS2, PLCG1, PLCG2,	1.45767E-16	5.38452E-3

						PTPN6, PVR, RAP1A, RET, SYK, PCDHGB7, PCDHGA5, PCDHB11, CDH26, VAV1, VAV2, ZAP70, PCDH19, PCDHB16, CDH22, DOK2, PCDHGB4, DCHS1, WASF2		
**Intermediate Filament Polymerization**	Cytoskeleton; Cytoskeleton Assembly; Cytoskeleton Assembly (Cell Process Pathways); Tissue Nonspecific	14	107	80	74	NUP50, KRT71, NUPL2, KRT74, DCTN1, DES, DYNC1I2, DYNC1H1, DYNC1LI2, DYNC1I1, NUP205, NUP210, SYNM, NUP35, AHCTF1, KRT25, DYNLL2, KRT72, GFAP, GLE1, KRT80, KRT24, KRT78, KRT1, KRT10, KRT4, KRT7, KRT5, KRT8, KRT9, KRT2, KRT3, KRT17, KRT31, KRT12, KRT19, KRT15, KRT13, KRT14, KRT16, KRT18, KRT33A, KRT32, KRT84, KRT35, KRT85, KRT82, KRT34, KRT86, LMNB1, DYNC2LI1, NUP54, NUP98, KRT76, KRT20, NDC1, NUP133, RANBP2,	7.44707E-14	5.06682E-3

						KRT79, KRT6C, KRT73, NUP43, KRT27, KRT77, KRT26, NUP85, RAE1, DYNC2H1, KRT37, SNX4, SYNC, SEH1L, NUP155, NUP93, KRT75, KRT39, NUP58, POM121, RGPD5, NUP153		
**Ca2+ Flux Regulation**	Nociception Overview Signaling; Nociception Overview Signaling (Nociception Pathways)	146	176	117	66	GNA13, BDKRB2, BDKRB1, ADCY3, ADCY8, ADCY7, ADCY9, ADCY5, ADCY6, ADCY1, ADCY2, ADORA3, ADRA1D, ADRB2, ADRA2C, ADRA1A, ADRA2A, ADRA1B, HRH3, CHRM1, CHRNA4, CHRM5, CHRM3, CHRNB2, CHRNA7, P2RX2, CALML4, BDNF, CACNA1E, CACNA1B, CACNA1C, CALCA, CALML3, CAMK2A, AKAP13, DRD4, DRD2, CNR1, GRIN3B, GRIN3A, CRH, CRHR1, PLCB1, EDN3, EDN1, EDNRB, PTK2B, GRIA1,	1.83868E-13	7.39523E-3

						GRIA3, GRIA4, GRIA2, GRIN2C, GRIN2B, GRIN2A, GRM5, GRM1, GRIN2D, GABBR1, GDNF, GNA15, GNA11, GNA12, GNAI2, GNAI1, GNAS, GNAQ, GNAI3, IRS1, ITPR1, HRH1, HRH2, HTR2B, HTR3A, HTR2C, HTR2A, HTR1E, HTR1F, ADCY4, CALML6, NGF, NTRK2, NTRK1, P2RY13, OPRL1, OPRK1, P2RX7, P2RX1, P2RX5, P2RX4, P2RY1, P2RY4, P2RY11, P2RY2, PRKACA, PRKCB, PRKD1, PRKCG, PDPK1, PLCG1, ADCY10, PTGER1, PTGIR, PTGFR, PTGER2, PTGER3, RET, SRC, TACR1, SHC1, HRH4, TRPV1, P2RY12, ARTN, GABBR2, CALCRL, RAMP1, P2RY14		
**G1/S Phase Transition**	Cell Cycle Regulation; Cell Cycle Regulation	45	154	103	66	PPP1R17, CCND1, AKT1, CDC25A,	2.94365E-12	6.51363E-3

	(Cell Process Pathways); Tissue Nonspecific					CDC34, CDK2, CDK4, CDK6, CDKN1B, UBE2Q2, UBE2C, CCNB1, CCNE1, CCNA2, CCNH, POLI, PPP1R13B, PSMB11, E2F3, E2F2, E2F1, E2F6, E2F4, PPP1R27, UBE2J2, PHLPP1, FOXM1, ERCC3, ERCC2, PPP1R21, PPP1R36, UBE2S, POLL, GTF2H3, GTF2H2, POLM, GTF2H1, GTF2H4, HELLS, UBE2U, E2F7, MNAT1, ATAD2, UBE2T, PPP1R35, POLE3, MYBL2, MYC, POLK, UBE2J1, PPP1R32, POLA1, POLD2, POLE2, POLH, UBE2R2, PPP1R2, PPP1R10, PPP1R7, PPP1R8, CDCA4, PSMA2, PSMA3, PSMB6, PSMA5, PSMA7, PSMB3, PSMA4, PSMA6, PSMB5, PSMB1, PSMB2, PSMB10, PSMB8, PSMB9, GTF2H2C, POLB, POLD1,		

						POLE, UBE2W, GTF2H2C_2, RB1, RBL2, PPP1R37, REV3L, UBE2Q1, PPP1R42, SKP1, SKP2, UBE2D1, UBE2L3, UBE2G1, UBE2N, PPP1R11, TFDP1, UBE2Z, CCNA1, RAD54L, CUL1, E2F8, CCNE2, UBE2L6, PPP1R26		
**G2/M Phase Transition**	Cell Cycle Regulation; Cell Cycle Regulation (Cell Process Pathways); Tissue Nonspecific	47	215	133	61	GADD45GIP1, NUP50, PPP1R17, PARP1, AKT1, CCNB3, CDK1, CDC25C, CDC25B, CDC34, CDC27, CDK2, CENPF, YWHAQ, UBE2Q2, UBE2C, NUPL2, CCNB1, PPP1R13B, NCAPH, PSMB11, E2F3, E2F2, E2F1, E2F6, E2F4, PPP1R27, NUP205, UBE2J2, NUP210, ANAPC16, PHLPP1, H1FOO, FOXM1, PPP1R21, NUP35, AHCTF1, SFN, PPP1R36, GSK3B, UBE2S, H1F0, HIST1H1D, HIST1H1E, HIST1H1B, HIST1H1C, HIST1H1T, HIST1H1A,	1.22915E-11	8.39434E-3

						UBE2U, GLE1, E2F7, UBE2T, ANAPC2, ANAPC4, PPP1R35, NUP54, NUP98, FZR1, ANAPC7, ANAPC5, UBE2J1, PPP1R32, ANAPC11, NEK2, UBE2R2, PPP1R2, PPP1R10, PPP1R7, PPP1R8, CDCA4, PSMA2, PSMA3, PSMB6, PSMA5, PSMA7, PSMB3, PSMA4, PSMA6, PSMB5, PSMB1, PSMB2, PSMB10, PSMB8, PSMB9, PLK1, NDC1, NUP133, UBE2W, RANBP2, RB1, PPP1R37, UBE2Q1, PPP1R42, SKP1, UBE2D1, UBE2L3, UBE2G1, UBE2N, WEE1, NCAPG, NUP43, YWHAB, YWHAH, PPP1R11, H1FNT, UBE2Z, YWHAZ, ANAPC1, NUP85, CDC16, CCNA1, BTRC, H1FX, CUL1, RAE1, E2F8, INCA1, CDC23, SEH1L, NUP155,		

						NUP93, PKMYT1, CCNB2, UBE2L6, KIF20A, SLK, DLGAP5, NUP58, PPP1R26, POM121, NCAPD2, RGPD5, NUP153		
**S/G2 Phase Transition**	Cell Cycle Regulation; Cell Cycle Regulation (Cell Process Pathways); Tissue Nonspecific	49	225	136	60	PPP1R17, TUBB3, TUBB4A, KAT5, TUBA3E, CDC25A, CDC34, CDC27, CDK2, CDKN1B, UBE2Q2, UBE2C, CCNB1, CCNA2, POLI, PPP1R13B, PSMB11, ORC6, ORC3, E2F3, E2F2, E2F1, E2F6, E2F4, PPP1R27, UBE2J2, ANAPC16, PHLPP1, H1FOO, PPP1R21, PPP1R36, TUBG2, UBE2S, POLL, POLM, H1F0, HIST1H1D, HIST1H1E, HIST1H1B, HIST1H1C, HIST1H1T, HIST1H1A, UBE2U, E2F7, MCM2, MCM3, MCM5, TUBD1, TUBE1, UBE2T, ANAPC2, RPA4, ANAPC4, NPM1, NPAT, PPP1R35, ORC5, ORC1, ORC4, ORC2, POLE3, TFDP3, TUBB, FZR1, POLK,	7.63618E-11	8.57990E-3

						ANAPC7, ANAPC5, UBE2J1, PPP1R32, ANAPC11, POLA1, POLD2, POLE2, POLH, UBE2R2, PPP1R2, PPP1R10, PPP1R7, PPP1R8, CDCA4, PSMA2, PSMA3, PSMB6, PSMA5, PSMA7, PSMB3, PSMA4, PSMA6, PSMB5, PSMB1, PSMB2, PSMB10, PSMB8, PSMB9, POLB, POLD1, POLE, RPA1, RPA3, RPA2, UBE2W, RB1, PPP1R37, REV3L, UBE2Q1, PPP1R42, SKP1, SKP2, TUBB2A, TUBA4A, TUBG1, UBE2D1, UBE2L3, UBE2G1, UBE2N, TUBB8, PPP1R11, TFDP1, H1FNT, UBE2Z, CDC45, CDC7, ANAPC1, CDC16, CCNA1, BTRC, H1FX, CUL1, E2F8, CDC23, TUBB1, UBE2L6, TUBB6, TUBA1C, CCP110, PPP1R26		

**Protein Folding**	Proteins Turnover; Proteins Turnover (Cell Process Pathways); Tissue Nonspecific	24	133	89	66	CCT6B, CCT8, DNAJB3, BAG1, TUBB3, TUBB4A, PFDN6, DNAJC14, CCT2, CCT4, CCT7, TUBA3E, DNAJC8, CCT5, CDC37, CCT6A, DNAJC16, PSMB11, HSPBP1, DNAJC9, DNAJC24, FKBP4, DNAJC21, SACS, DNAJC25, DNAJB5, TUBG2, DNAJC2, HSCB, DNAJB7, HSPA1A, DNAJB2, HSP90AA1, HSPA8, DNAJB1, DNAJC4, TUBD1, TUBE1, HSPA14, DNAJC15, DNAJB8, DNAJB11, DNAJC10, DNAJC18, TUBB, DNAJC28, DNAJC3, PSMA2, PSMA3, PSMB6, PSMA5, PSMA7, PSMB3, PSMA4, PSMA6, PSMB5, PSMB1, PSMB2, PSMB10, PSMB8, PSMB9, PFDN5, PFDN2, PFDN4, DNAJC5G, DNAJC11, UNC45A,	8.60813E-11	5.63077E-3

						DNAJC17, DNAJC12, DNAJC7, TUBB2A, TUBA4A, TUBG1, VBP1, TUBB8, TCP1, HSP90B1, CCT3, DNAJB13, DNAJC1, DNAJC22, DNAJC5, TUBB1, DNAJB6, TUBB6, TUBA1C, STUB1, DNAJC6, TOMM70		
**Golgi to Endosome Transport**	Endomembrane System; Tissue Nonspecific; Vesicular Transport; Vesicular Transport (Cell Process Pathways)	59	128	85	66	YKT6, ARF1, AP4B1, AP2A2, AP1G1, AP2A1, AP2B1, COG5, AP1B1, SYNRG, VPS26B, COG2, TRAPPC8, AP1S1, AP3S1, AP2M1, CALML4, AP3M2, BNIP1, CALML3, AP4S1, AP4E1, DNM1, DNM2, TRAPPC5, GGA2, GGA1, RASGRP3, EPS15, COG4, AP1S3, VTI1A, AP3M1, TRAPPC3, GOLGA4, GOLGA1, IGF2R, TRAPPC12, CALML6, M6PR, NSF, FURIN, BET1L, TSNARE1, TRAPPC4, PRKCD, AFTPH,	4.14454E-10	5.37804E-3

						PACS1, VPS35, USE1, RAB5A, SNX2, TRAPPC2, SLC18A3, SNX1, SMOC2, COG6, TRAPPC10, TRAPPC1, AP3B2, TRAPPC6A, PICALM, EEA1, NAPA, AP1G2, AP1M1, AP1S2, AP3D1, AP3B1, STX16, VAMP4, SNX4, GOSR1, VPS26A, GOSR2, COG3, CLINT1, AP4M1, RAB9A, AP1M2, COG8, ARFRP1, STX6, PLIN3, SGIP1		
**Endosomal Recycling**	Endomembrane System; Tissue Nonspecific; Vesicular Transport; Vesicular Transport (Cell Process Pathways)	50	108	74	68	MVB12B, ARF1, ARF6, AP4B1, RAB10, AP2A2, AP1G1, AP2A1, AP2B1, AP1B1, SNF8, SYNRG, RAB11FIP2, EPN2, AP1S1, AP3S1, AP2M1, EHD1, AP3M2, CHMP4C, RAB35, AP4S1, PIP5K1C, AP4E1, DNM1, DNM2, RAB11FIP5, VPS37A, PIP5KL1, EPS15, CHMP4B, AP1S3, AP3M1, VPS37D,	6.44160E-10	4.68473E-3

						VPS36, VPS28, EPN1, LDLR, EHD4, EPN3, VPS37C, PLD1, AFTPH, RAB5A, CHMP1B, RAB22A, PARD3, SLC18A3, TSG101, RBSN, TF, TFRC, AP3B2, PIP5K1A, PIP5K1B, EEA1, RAB11A, AP1G2, AP1M1, AP1S2, AP3D1, RAB11FIP1, CHMP6, AP3B1, VPS37B, SNX4, AP4M1, REPS2, VPS25, AP1M2, RAB11FIP3, IST1, SGIP1, PDCD6IP		
**Kinetochore Assembly**	Cell Division; Cell Division (Cell Process Pathways); Nucleus; Tissue Nonspecific	48	90	64	71	DCTN6, STAG2, CENPL, NDC80, DCTN2, DCTN3, CDC20, CDC27, OIP5, CENPE, CENPB, CENPC, CENPF, KIF2C, BUB1B, ZWINT, DAXX, DCTN1, DYNC1I2, DYNC1H1, DYNC1LI2, DYNC1I1, ANAPC16, CENPI, DYNLL2, INCENP, MXD1, MAD2L1, DCTN4, ANAPC2, ANAPC4, DYNC2LI1, FZR1,	8.44242E-10	4.05371E-3

						ANAPC7, ANAPC5, ANAPC11, ZWILCH, CDCA8, PLK1, CENPN, ACTR10, RAD21, RANGAP1, CASC5, SPC25, TTK, XPO1, CENPM, CENPO, MAD1L1, ANAPC1, CENPH, CDC16, CENPT, DYNC2H1, CDC23, SNX4, NUF2, ZW10, BUB3, AURKB, ACTR1B, ACTR1A, STAG1		

PATHWAY STUDIO

Entity List Summary Analysis for CD4+ T cells less than 300

generated by **Pathway Studio**

Group: CD4 less than 300

# of Entities: 2115

This summary report contains 1 result with 10 pathways.

Table of content

Top Pathways

- Biological Function
  - Neutrophil Chemotaxis
  - Vascular Motility
  - Platelet Activation via GPCR Signaling
  - Insulin Influence on Protein Synthesis
  - mTOR Signaling Overview
  - EDNRA/B -> Vascular Motility
  - Proplatelet Maturation
  - Natural Killer Cell Activation through ITAM-Containing Receptors
  - Taste Sensor Receptors Activates mTOR Signaling (Rodent Model)
  - Natural Killer Cell Activation

Top Pathways

Biological Function

Name: Pathways/Groups similar to CD4 less than 300

Name	Parent Folder	# of Entities	Expanded # of Entities	Overlap	Percent Overlap	Overlapping Entities	p-value	Jaccard similarity
**Neutrophil Chemotaxis**	Nervous Tissue; Neutrophils Activation; Neutrophils Activation (Inflammation Pathways)	81	185	23	12	RASL10A, PPBP, OC90, PRKACB, PRKCA, PRKACG, MAPK3, DOCK3, CXCL6, MYL6B, PDPK1, KRAS, CYBA, CDC42, DOCK5, CXCR2, RAC1, MYL2, ERAS, MYLK, RERGL, JMJD7-PLA2G4B, RAB6B	6.05950E-5	1.01010E-2
**Vascular Motility**	Nociception Overview Signaling; Nociception Overview Signaling (Nociception Pathways)	117	168	21	12	ARHGEF12, RASL10A, PRKACB, PRKCA, PRKACG, MAPK3, ARHGEF1, PDPK1, KRAS, ADORA2A, GNAS, GNAO1, CALML5, EDNRA, ERAS, MYLK, HTR1B, HTR1A, RERGL, HTR6, RAB6B	1.16492E-4	9.28382E-3
**Platelet Activation via GPCR Signaling**	Cardiovascular System; Fluid Connective Tissue; Hemostasis Reaction; Hemostasis Reaction (Inflammation Pathways)	81	154	19	12	RASL10A, OC90, PRKACB, PRKCA, PRKACG, MAPK3, PDPK1, KRAS, ADORA2A, GNAS, GNAO1, CDC42, F2RL3, RAC1,	2.89264E-4	8.44444E-3

						ERAS, MYLK, RERGL, JMJD7-PLA2G4B, RAB6B		
**Insulin Influence on Protein Synthesis**	Digestive System; Metabolism Regulation	37	65	11	16	RASL10A, MAPK3, PDPK1, KRAS, RPTOR, EIF2S3, EIF4EBP1, ERAS, RERGL, RAB6B, RHEB	3.85208E-4	5.07146E-3
**mTOR Signaling Overview**	Signal Transduction Pathways; Tissue Nonspecific; mTOR Signaling and Aging; mTOR targets	112	297	29	9	RASL10A, DAP, PRKACB, PRKCA, PRKACG, PSMB4, PSMB7, PDPK1, KRAS, PSMA8, RRAGD, UBE2A, UBE2E1, CALML5, UBE2E2, UBE2D2, UBE2QL1, UBE2G2, ATG14, DDIT4L, YY1, YWHAE, RPTOR, TBC1D7, EIF4EBP1, ERAS, RERGL, RAB6B, RHEB	5.36381E-4	1.21695E-2
**EDNRA/B -> Vascular Motility**	Endothelin Receptors; Endothelin Receptors (Nociception Pathways); Epithelium	60	114	15	13	ARHGEF12, RASL10A, PRKACB, PRKCA, PRKACG, MAPK3, ARHGEF1, KRAS, CDC42, CALML5, EDNRA, ERAS, MYLK, RERGL, RAB6B	6.26638E-4	6.77507E-3
**Proplatelet Maturation**	Cardiovascular System; Fluid Connective Tissue; Hemostasis Reaction; Hemostasis Reaction	100	178	20	11	GP1BA, GP9, RASL10A, PPBP, PRKACB, PRKCA, PRKACG, MAPK3, PDPK1,	6.93418E-4	8.79894E-3

	(Inflammation Pathways)					KRAS, ZBTB16, CSF2RA, GNAS, IFNGR2, IL6ST, IL11, CCL5, ERAS, RERGL, RAB6B		
**Natural Killer Cell Activation through ITAM-Containing Receptors**	Lymphatic System; Natural Killer Cell Receptors Signaling; Natural Killer Cell Receptors Signaling (Immunological Pathways)	86	153	18	11	RASL10A, PPP3CB, MAPK3, HLA-A, HLA-B, KIR2DS1, PDPK1, KIR2DS5, KLRD1, KRAS, CDC42, MZT2B, LAT2, HLA-C, RAC1, ERAS, RERGL, RAB6B	7.38095E-4	8.00000E-3
**Taste Sensor Receptors Activates mTOR Signaling (Rodent Model)**	Metabolism Regulation	56	97	13	13	RASL10A, TAS2R1, PRKACB, PRKACG, KRAS, TRPM5, SLC2A2, RPTOR, EIF4EBP1, ERAS, RERGL, RAB6B, RHEB	1.19456E-3	5.91178E-3
**Natural Killer Cell Activation**	Lymphatic System; Self Tolerance; Self Tolerance (Immunological Pathways)	82	136	16	11	RASL10A, PPP3CB, STAT5A, MAPK3, KRAS, IFNGR2, CD48, CDC42, CALML5, IL2RA, IL2RB, RAC1, ERAS, RERGL, RAB6B, CD2	1.43047E-3	7.15884E-3

PATHWAY STUDIO

Entity List Summary Analysis for Low VL, Less than 200 copies

generated by **Pathway Studio**

Summary

Group: Low VL

# of Entities: 15028

This summary report contains 1 result with 10 pathways.

Table of content

Top Pathways

Biological Function

Intermediate Filament Polymerization

Natural Killer Cell Inhibitory Receptor Signaling

Golgi to Endosome Transport

Ca2+ Flux Regulation

HRH1/3 -> Synaptic Transmission

Vascular Motility

Endosomal Recycling

G1/S Phase Transition

Golgi Transport

G2/M Phase Transition

PATHWAY STUDIO

Top Pathways

Biological Function

Name: Pathways/Groups similar to Low VL

Name	Parent Folder	# of Entities	Expanded # of Entities	Overlap	Percent Overlap	Overlapping Entities	p-value	Jaccard similarity
**Intermediate Filament Polymerization**	Cytoskeleton; Cytoskeleton Assembly; Cytoskeleton Assembly (Cell Process Pathways); Tissue Nonspecific	14	107	81	75	NUP50, KRT71, KRT74, DCTN1, DES, KRT40, DYNC1I2, DYNC1H1, DYNC1LI2, NUP205, NUP210, SYNM, NUP35, AHCTF1, KRT25, DYNLL2, KRT72, GFAP, GLE1, KRT80, KRT24, KRT78, DYNC1LI1, KRT1, KRT6A, KRT10, KRT7, KRT5, KRT8, KRT9, KRT2, KRT3, KRT17, KRT31, KRT12, KRT19, KRT15, KRT13, KRT14, KRT16, KRT18, KRT33A, KRT33B, KRT32, KRT84, KRT35, KRT85, KRT82, KRT34, KRT86, LMNB1, DYNC2LI1, NUP54, NUP98, KRT76, PRPH, KRT20, NDC1, NUP133, RANBP2,	1.44476E-15	5.38099E-3

						KRT79, KRT73, NUP43, DYNLT1, KRT27, KRT77, NUP37, KRT26, RAE1, DYNC2H1, KRT38, SNX4, SYNC, SEH1L, NUP155, NUP93, KRT75, NUP58, POM121, RGPD5, NUP153		
**Natural Killer Cell Inhibitory Receptor Signaling**	Lymphatic System; Natural Killer Cell Receptors Signaling; Natural Killer Cell Receptors Signaling (Immunological Pathways)	62	109	79	72	LILRB1, ABL1, VAV3, CD300A, CDH9, CDH5, CDH1, CDH2, CDH8, CDH4, CDH7, CDH3, CDH10, CDH11, CDH16, CDH18, CDH15, CDH17, CDH13, CDH12, CBL, DOK1, COL1A1, COL1A2, CRK, CSK, PCDHGA12, PCDHB5, FAT1, FAT2, SIGLEC9, HLA-A, HLA-B, FYN, LAT, SIGLEC7, INPP5D, HLA-E, HLA-G, CDH19, KIR2DL1, KIR3DL1, KIR3DL2, CLEC2D, KLRC1, KLRB1, LAIR1, LCK, LCP2, LYN, PAK1, PCDH8, TIGIT, MAP2K1, PCDH1, PCDH7, PCDHGC3, DCHS2, PLCG1, PLCG2,	1.89957E-13	5.24673E-3

						PTPN6, PTPN11, PVR, RAP1A, RET, SYK, PCDHGB7, PCDHB11, CDH26, VAV1, VAV2, CDH23, PCDHB16, CDH22, CDH24, DOK2, PCDHGB4, DCHS1, WASF2		
**Golgi to Endosome Transport**	Endomembrane System; Tissue Nonspecific; Vesicular Transport; Vesicular Transport (Cell Process Pathways)	59	128	86	67	TGOLN2, YKT6, ARF1, AP4B1, AP2A2, AP1G1, AP2A1, AP2B1, COG5, AP1B1, SYNRG, VPS26B, COG2, TRAPPC8, AP3S1, CALML4, COG7, AP3M2, BNIP1, CALML3, AP4S1, TRAPPC6B, AP4E1, DNM2, TRAPPC5, GGA2, CPD, GGA1, RASGRP3, EPS15, COG4, VTI1A, AP3M1, TRAPPC3, GOLGA4, GOLGA1, IGF2R, TRAPPC12, CALML6, M6PR, VPS29, CALML5, NSF, FURIN, BET1L, TSNARE1, TRAPPC4, PRKCD, AFTPH, PACS1, USE1, RAB5A, RHEB, SNX2, TRAPPC3L, TRAPPC2,	1.42842E-11	5.70708E-3

						SLC18A3, SMOC2, COG6, TRAPPC10, AP3B2, TRAPPC6A, PICALM, EEA1, AP1G2, AP1M1, AP3D1, AP3B1, STX16, VAMP4, SNX4, GOSR1, VPS26A, GOSR2, COG3, TRAPPC9, CLINT1, AP4M1, RAB9A, COG1, AP1M2, COG8, ARFRP1, STX6, PLIN3, SGIP1		
**Ca2+ Flux Regulation**	Nociception Overview Signaling; Nociception Overview Signaling (Nociception Pathways)	146	176	110	62	GNA13, BDKRB2, ADCY3, ADCY8, ADCY7, ADCY9, ADCY5, ADCY6, ADCY1, ADCY2, ADRA1D, ADRB2, ADRA2C, ADRA1A, ADRA2A, HRH3, CHRM1, CHRNA4, CHRM5, CHRM3, CHRNB2, CHRNA7, P2RX2, CALML4, BDNF, CACNA1E, CACNA1B, CACNA1C, CALCA, CALML3, AKAP13, DRD4, DRD2, CNR1, GRIN3B, GRIN3A, CRHR1, PLCB1, EDN1,	2.39216E-11	7.28815E-3

						EDNRB, PTK2B, GRIA1, GRIA3, GRIA4, GRIA2, GRIN2C, GRIN2B, GRIN1, GRIN2A, GRM1, GRIN2D, GABBR1, GNA15, GNA11, GNA12, GNAI1, GNAS, GNAQ, GNAO1, IRS1, ITPR1, HRH1, HRH2, HTR1B, HTR1A, HTR2B, HTR3A, HTR2C, HTR1D, HTR1E, HTR1F, ADCY4, CALML6, NGF, CALML5, NTRK2, NTRK1, P2RY13, OPRK1, P2RX7, P2RX1, P2RX5, P2RY6, P2RY1, P2RY4, P2RY2, PRKACA, PRKACB, PRKCA, PRKCB, PRKACG, PRKD1, PRKCG, PDPK1, PLCG1, ADCY10, PTGIR, PTGFR, PTGER3, RET, SRC, TACR1, SHC1, HRH4, TRPV1, P2RY12, GABBR2, CALCRL, RAMP1, P2RY14		

**HRH1/3 -> Synaptic Transmission**	Histamine Receptor; Histamine Receptor (Nociception Pathways); Nervous Tissue; Neuronal System	18	60	47	78	SYT8, STX19, SYT12, ADCY3, ADCY8, ADCY7, ADCY9, ADCY5, ADCY6, ADCY1, ADCY2, HRH3, STX1B, CACNA1C, SYT11, SYT2, STX2, SYT9, GNAI1, GNAQ, ITPR1, HRH1, ADCY4, SYT17, PRKACA, PRKACB, STX17, PRKACG, SYT14, PLCB3, ADCY10, STX4, STX1A, VAMP2, SYT1, SYT4, SYT5, SYT13, SYT10, STX7, SYT7, STX11, STX16, STX6, SYT16, SYT15, SYT3	1.78779E-10	3.12500E-3
**Vascular Motility**	Nociception Overview Signaling; Nociception Overview Signaling (Nociception Pathways)	117	168	103	61	RASL10A, GNA13, RASL10B, BDKRB2, ACTA2, MYL9, ADCY3, ADCY8, ADCY7, ADCY9, ADCY5, ADCY6, ADCY1, ADCY2, ADORA2A, RERG, ADORA2B, ADRA1D, ADRB3, ADRB2, ADRA2C, ADRA1A, AKT1, RRAS2, MRAS, CHRM2, CHRM1, CHRM3, CALML4, BRAF, IFT27,	5.12771E-10	6.82481E-3

						CACNA1B, CACNA1C, CALCA, CALML3, AKAP13, ARHGEF12, CNR1, CNR2, PLCB1, EDN1, EDNRB, GNA15, GNA11, GNA12, GNAI1, GNAS, GNAQ, GNAO1, ITPR1, HRH1, HRH2, HTR1B, HTR1A, HTR4, HTR1D, HTR1E, HTR1F, HTR6, HTR7, ADCY4, KRAS, CALML6, NOS3, NOS1, CALML5, NRAS, RASL12, MYLK, PPP1CB, PRKACA, PRKACB, PRKCA, PRKCB, PRKACG, PRKD1, PRKCG, MAPK3, MAPK1, PRKG1, PRKG2, MAP2K1, MAP2K2, PDE4A, PDPK1, ROCK1, RRAS, ADCY10, PTGER4, PTGIR, PTGFR, PTGER3, RAP1A, RASGRF1, RIT2, RIT1, SRC, TRPV1, PDE5A, ARHGEF1, FARP1, CALCRL, RAMP1		

**Endosomal Recycling**	Endomembrane System; Tissue Nonspecific; Vesicular Transport; Vesicular Transport (Cell Process Pathways)	50	108	72	66	MVB12B, ARF1, ARF6, AP4B1, RAB10, CHMP7, AP2A2, AP1G1, AP2A1, AP2B1, AP1B1, SNF8, SYNRG, RAB11FIP2, AP3S1, EHD1, AP3M2, CHMP4C, RAB35, AP4S1, PIP5K1C, AP4E1, DNM2, RAB11FIP5, VPS37A, PIP5KL1, EPS15, CHMP4B, CHMP2B, AP3M1, VPS37D, VPS36, VPS28, CHMP4A, EPN1, LDLR, EHD4, CHMP3, EPN3, VPS37C, PIK3CG, PLD1, AFTPH, RPS27A, RAB5A, RAB22A, PARD3, SLC18A3, TSG101, RBSN, TF, TFRC, AP3B2, PIP5K1A, PIP5K1B, EEA1, RAB11A, AP1G2, AP1M1, AP3D1, RAB11FIP1, CHMP6, AP3B1, VPS37B, SNX4, AP4M1, REPS2, AP1M2, RAB11FIP3, IST1, SGIP1, PDCD6IP	1.12436E-9	4.77992E-3

**G1/S Phase Transition**	Cell Cycle Regulation; Cell Cycle Regulation (Cell Process Pathways); Tissue Nonspecific	45	154	95	61	PPP1R17, AKT1, CDC25A, CDK2, CDK4, CDK6, CDKN1C, CDKN1B, CKS1B, UBE2Q2, UBE2C, CCNE1, CCNA2, CCNH, POLI, PPP1R13B, PSMB11, E2F3, E2F2, E2F1, E2F6, E2F4, UBE2J2, PHLPP1, FOXM1, UBE2QL1, ERCC3, ERCC2, PPP1R21, PPP1R36, UBE2S, POLL, GTF2H3, GTF2H1, GTF2H4, HELLS, UBE2K, UBE2U, PSMA8, E2F7, MNAT1, ATAD2, UBE2T, PPP1R35, POLE3, MYBL2, MYC, POLK, UBE2J1, PPP1R32, UBE2D4, POLA1, POLD2, POLE2, POLH, UBE2R2, PPP1R2, PPP1R10, PPP1R7, PPP1R8, CDCA4, PSMA1, PSMA2, PSMA3, PSMB4, PSMA7, PSMB3, PSMA4, PSMA6, PSMB5, PSMB1, PSMB8, PSMB9, GTF2H2C,	1.51738E-9	6.29723E-3

						POLB, POLE, UBE2W, GTF2H2C_2, RB1, RBL2, PPP1R37, REV3L, UBE2Q1, PPP1R42, SKP1, SKP2, UBE2G1, TFDP1, UBE2Z, RAD54L, CUL1, E2F8, CCNE2, PPP1R26, RBX1		
**Golgi Transport**	Endomembrane System; Tissue Nonspecific; Vesicular Transport; Vesicular Transport (Cell Process Pathways)	36	90	62	68	ARCN1, ARF1, ATP2B1, ATP2B2, SEC24A, ATP2B4, ATP2B3, SEC24B, COG5, SEC23A, COPE, COG2, COPG1, SEC31A, TRAPPC8, COG7, KDELR1, BNIP1, KDELR2, TRAPPC6B, TRAPPC5, COPB1, COPA, COG4, SEC31B, GAPDH, COPG2, ATP2C1, GLT1D1, TRAPPC3, MANEAL, TRAPPC12, COPZ2, TSNARE1, ERGIC2, TRAPPC4, ERGIC3, ARFGAP1, USE1, RAB2A, RAB6A, RAB1A, ERGIC1, TRAPPC3L, SEC13, TRAPPC2, COG6, TRAPPC10, TRAPPC6A, GBF1, GOSR1,	2.21258E-9	4.11823E-3

						GOSR2, SEC24C, COG3, TRAPPC9, COPB2, COG1, COG8, PREB, SEC24D, ATP2C2, SEC16A		
**G2/M Phase Transition**	Cell Cycle Regulation; Cell Cycle Regulation (Cell Process Pathways); Tissue Nonspecific	47	215	123	57	GADD45GIP1, NUP50, PPP1R17, ANAPC10, PARP1, AKT1, CCNB3, CDK1, CDC25C, CDC25B, CDC27, CDK2, CENPF, YWHAQ, UBE2Q2, UBE2C, PPP1R13B, NCAPH, PSMB11, E2F3, E2F2, E2F1, E2F6, E2F4, NUP205, UBE2J2, NUP210, PHLPP1, H1FOO, FOXM1, UBE2QL1, FBXO5, FBXO7, PPP1R21, NUP35, AHCTF1, SFN, PPP1R36, GSK3B, UBE2S, H1F0, HIST1H1E, HIST1H1B, HIST1H1T, HIST1H1A, UBE2K, UBE2U, GLE1, PSMA8, E2F7, UBE2T, ANAPC2, PPP1R35, NUP54, NUP98, FZR1, ANAPC5, UBE2J1, PPP1R32, NEK2, UBE2D4, UBE2R2,	5.32437E-9	8.13546E-3

						PPP1R2, PPP1R10, PPP1R7, PPP1R8, CDCA4, PSMA1, PSMA2, PSMA3, PSMB4, PSMA7, PSMB3, PSMA4, PSMA6, PSMB5, PSMB1, PSMB8, PSMB9, NDC1, NUP133, UBE2W, RANBP2, RB1, PPP1R37, UBE2Q1, PPP1R42, SKP1, UBE2G1, WEE1, NCAPG, NUP43, YWHAE, YWHAH, H1FNT, UBE2Z, NUP37, YWHAZ, YWHAG, ANAPC1, CDC16, BTRC, H1FX, CUL1, RAE1, E2F8, INCA1, CDC23, SEH1L, NUP155, NUP93, PKMYT1, CCNB2, KIF20A, SLK, DLGAP5, NUP58, PPP1R26, POM121, NCAPD2, RGPD5, NUP153, RBX1		

PATHWAY STUDIO

Entity List Summary Analysis for High VL, Greater Than 200 Copies

generated by **Pathway Studio**

Summary

Group: High VL

# of Entities: 2486

This summary report contains 1 result with 10 pathways.

Table of content

Top Pathways

- Biological Function
  - Metaphase/Anaphase Phase Transition
  - S/G2 Phase Transition
  - Spindle Assembly
  - Kinetochore Assembly
  - Cell Cycle Overiew
  - Natural Killer Cell Activation
  - Histone Ubiquitylation
  - Eosinophil Survival by Cytokine Signaling
  - Protein Folding
  - G2/M Phase Transition

Top Pathways

Biological Function

Name: Pathways/Groups similar to High VL

Name	Parent Folder	# of Entities	Expanded # of Entities	Overlap	Percent Overlap	Overlapping Entities	p-value	Jaccard similarity
**Metaphase/Anaphase Phase Transition**	Cell Cycle Regulation; Cell Cycle Regulation (Cell Process Pathways); Tissue Nonspecific	16	60	15	25	STAG2, AURKA, PSMB6, PSMA5, PSMB2, PSMB10, AURKB, PTTG1, ANAPC4, ANAPC16, PLK1, RAD21, ANAPC7, ANAPC11, CCNB1	2.04093E-6	5.92183E-3
**S/G2 Phase Transition**	Cell Cycle Regulation; Cell Cycle Regulation (Cell Process Pathways); Tissue Nonspecific	49	225	33	14	CDT1, HIST1H1D, H2AFX, HIST1H1C, PSMB6, PSMA5, PSMB2, PSMB10, E2F5, PPP1R27, TUBB4A, UBE2L6, ANAPC4, ANAPC16, POLD1, CDC6, CDC34, NPM1, CDKN1A, UBE2D1, UBE2D3, UBE2H, UBE2G2, TUBA8, CCNA1, ORC1, ORC4, CCP110, PPP1R11, TUBB, ANAPC7, ANAPC11, CCNB1	2.05504E-6	1.23134E-2
**Spindle Assembly**	Cell Division; Cell Division (Cell Process	29	71	15	21	STAG2, DYNC1I1, DYNLRB2,	1.87856E-5	5.89623E-3

	Pathways); Nucleus; Tissue Nonspecific					DYNLRB1, AURKB, PTTG1, ANAPC4, ANAPC16, PLK1, DCTN3, ACTR1A, DYNLT3, RAD21, ANAPC7, ANAPC11		
**Kinetochore Assembly**	Cell Division; Cell Division (Cell Process Pathways); Nucleus; Tissue Nonspecific	48	90	16	17	STAG2, CENPO, DYNC1I1, SPC24, DYNLRB2, DYNLRB1, AURKB, ANAPC4, ANAPC16, PLK1, DCTN3, ACTR1A, DYNLT3, RAD21, ANAPC7, ANAPC11	9.31829E -5	6.24512E -3
**Cell Cycle Overiew**	Cell Cycle Regulation; Cell Cycle Regulation (Cell Process Pathways); Tissue Nonspecific	140	304	35	11	POLR2F, STAG2, H2AFX, CCND1, XRCC6, GTF2H5, PLK1, CDC6, CDKN1A, CDKN2C, CCNA1, DBF4, BRCA1, RAD21, CCNB1, CCNC, CDT1, GADD45A, AURKA, E2F5, AURKB, PTTG1, ANAPC4, ANAPC16, NPM1, ORC1, ORC4, POLR2J2, YWHAB, TGFB2, TGFBR1, POLR2J3, GADD45B, ANAPC7, ANAPC11	1.97162E -4	1.26950E -2
**Natural Killer Cell Activation**	Lymphatic System; Self Tolerance;	82	136	19	13	RASL11B, GRB2, STAT5A,	5.68147E -4	7.29367E -3

	Self Tolerance (Immunological Pathways)					ULBP2, ULBP1, BAIAP2, IFNG, CDC42, ARPC3, ARPC2, IL2RA, RASL11A, RAF1, CALM1, CALM2, CALM3, ICAM1, RAB6B, CD247		
**Histone Ubiquitylation**	Histone Modification; Histone Modification (Cell Process Pathways); Nucleus; Tissue Nonspecific	21	120	17	14	POLR2F, H2AFX, H3F3B, DZIP3, H2BFM, UBE2L6, CDC34, UBA1, UBE2D1, UBE2D3, UBE2H, UBE2G2, POLR2J2, CDC73, BRCA1, POLR2J3, H2AFV	9.30318E-4	6.56117E-3
**Eosinophil Survival by Cytokine Signaling**	Eosinophils Activation; Eosinophils Activation (Inflammation Pathways); Fluid Connective Tissue; Lymphatic System	86	188	23	12	RASL11B, JDP2, GRB2, STAT5A, MAPK8, BCL2, CCL2, SDCBP, CSF2, IL3, IL5, RPS6KA2, IL15, IL15RA, IL27, YWHAB, RASL11A, MYD88, RAF1, TNFRSF1A, ICAM1, RAB6B, RIPK1	1.06027E-3	8.66943E-3
**Protein Folding**	Proteins Turnover; Proteins Turnover (Cell Process Pathways); Tissue Nonspecific	24	133	18	13	ST13, PSMB6, PSMA5, HSPA14, PSMB2, PSMB10, DNAJC5, TUBB4A, PFDN5, PFDN4, CCT4, TUBA8,	1.14670E-3	6.91510E-3

						VBP1, TUBB, HSCB, DNAJB7, DNAJB5, DNAJC4		
**G2/M Phase Transition**	Cell Cycle Regulation; Cell Cycle Regulation (Cell Process Pathways); Tissue Nonspecific	47	215	25	11	AURKA, HIST1H1D, HIST1H1C, PSMB6, PSMA5, PSMB2, PSMB10, E2F5, PPP1R27, UBE2L6, ANAPC4, ANAPC16, PLK1, NUP85, CDC34, UBE2D1, UBE2D3, UBE2H, UBE2G2, CCNA1, YWHAB, PPP1R11, ANAPC7, ANAPC11, CCNB1	1.36679E -3	9.33532E -3
